# PD-1 expression on mouse intratumoral NK cells and its effects on NK cell phenotype

**DOI:** 10.1016/j.isci.2022.105137

**Published:** 2022-09-16

**Authors:** Arnika K. Wagner, Nadir Kadri, Chris Tibbitt, Koen van de Ven, Sunitha Bagawath-Singh, Denys Oliinyk, Eric LeGresley, Nicole Campbell, Stephanie Trittel, Peggy Riese, Ulf Ribacke, Tatyana Sandalova, Adnane Achour, Klas Kärre, Benedict J. Chambers

**Affiliations:** 1 Center for Hematology and Regenerative Medicine, Department of Medicine, Karolinska Institutet, Huddinge, Stockholm, Sweden; 2 Center for Infectious Medicine, Department of Medicine, Karolinska Institutet, Huddinge, Stockholm, Sweden; 3 Science for Life Laboratory, Department of Medicine Solna, Karolinska Institute, and Division of Infectious Diseases, Karolinska University Hospital, Stockholm, Sweden; 4 Department of Microbiology, Tumor and Cell Biology, Karolinska Institutet, Stockholm, Sweden; 5 Centre for Infectious Disease Control, National Institute for Public Health and the Environment (RIVM), Bilthoven, the Netherlands; 6 Department of Vaccinology and Applied Microbiology, Helmholtz Centre for Infection Research, Braunschweig, Germany

**Keywords:** Immunology, Components of the immune system, Cancer, Transcriptomics

## Abstract

Although PD-1 was shown to be a hallmark of T cells exhaustion, controversial studies have been reported on the role of PD-1 on NK cells. Here, we found by flow cytometry and single cell RNA sequencing analysis that PD-1 can be expressed on MHC class I-deficient tumor-infiltrating NK cells *in vivo*. We also demonstrate distinct alterations in the phenotype of *PD-1*-deficient NK cells and a more mature phenotype which might reduce their capacity to migrate and kill *in vivo.* Tumor-infiltrating NK cells that express PD-1 were highly associated with the expression of CXCR6. Furthermore, our results demonstrate that PD-L1 molecules in membranes of *PD-1*-deficient NK cells migrate faster than in NK cells from wild-type mice, suggesting that PD-1 and PD-L1 form *cis* interactions with each other on NK cells. These data demonstrate that there may be a role for the PD-1/PD-L1 axis in tumor-infiltrating NK cells *in vivo*.

## Highlights

• NK cells from PD-1 deficient mice have a more mature phenotype
• Elimination of MHC-I-deficient cells is impaired in *PD-1*^−/−^ mice
• PD-1 expression on NK cells is associated with surface expression of CXCR6
• PD-1/PD-L1 interactions on NK cells may occur in cis

## Graphical abstract

**Figure:**
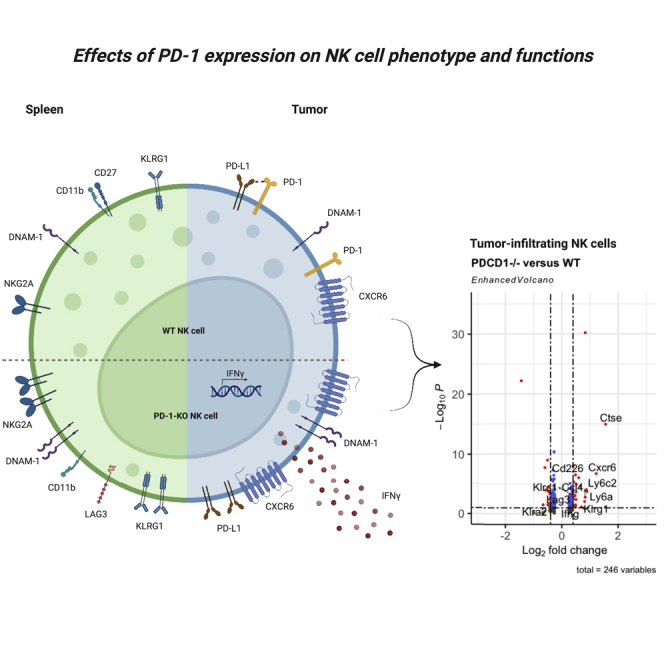

## Introduction

Natural killer (NK) cells are innate lymphoid cells (ILCs) that can kill tumor cells, stressed or virus infected cells (Biron, 1997; Herberman et al., 1975; Kiessling et al., 1975). NK cell activation is dependent on signals from activating and inhibitory receptors as well as pro-inflammatory cytokines (Kadri et al., 2016). Activating NK cell receptors can recognize stress-induced molecules, which induce phosphorylation events that may culminate in the release of cytotoxic granules and cytokines (Lanier, 2005). Healthy cells are protected from killing by NK cells because of the expression of self-MHC class I molecules (MHC-I) on their surface which act as ligands for dominant inhibitory receptors (Kärre et al., 1986). These receptors include killer cell immunoglobulin-like receptors (KIRs) in humans, Ly49 molecules in mouse and NKG2A in both species (Lanier, 2008). Engagement of inhibitory receptors results in recruitment of phosphates such as SHP-1, SHP-2 and SHIP-1, and dephosphorylation of signaling molecules which prevents NK cell-mediated killing.

NK cells express also non-MHC-I recognizing inhibitory receptors, known as checkpoint receptors, including TIGIT, LAG-3, CTLA-4, and PD-1. Clinically, antibodies against CTLA-4 and PD-l (or its ligand PD-L1) have been found to be relatively successful in therapy to certain forms of solid cancer (Pardoll, 2012; Seidel et al., 2018). Similar to KIR and Ly49 molecules, several checkpoint receptors found on NK cells can recruit and activate phosphatases (Chiossone et al., 2017). Several studies have identified subsets of NK cells expressing PD-1 (Beldi-Ferchiou et al., 2016; Benson et al., 2010; Hsu et al., 2018; Quatrini et al., 2018) in various disease settings but also in healthy individuals (Pesce et al., 2017). Furthermore, there is accumulating evidence that NK cells participate in the therapeutic effects of antibodies against PD-1 or PD-L1, especially toward tumors with low MHC-I expression (Ansell et al., 2015; Benson et al., 2010; Bezman et al., 2017; Guo et al., 2016; Hsu et al., 2018; Huang et al., 2018; Liu et al., 2017; Seo et al., 2018).

Recently, PD-1 expression was detected early in the development of some ILC subsets, which was thought to play a role in the development of ILC responses and raised the possibility that ILC subsets could be depleted with anti-PD-1 antibody (Yu et al., 2016). These data raised the question of if and how PD-1 is involved in NK cell development and education, and how a chronic lack of PD-1 expression may affect NK cell functions. In the present study, we examined the role of PD-1 in NK cell function using NK cells from *PD-1*-deficient mice and the potential role of PD-1/PD-L1 interactions in controlling NK cell activity.

## Results

### NK cell phenotype and population sizes are affected in *PD-1*^−/−^ mice compared to wild-type mice

**Figure 1:**
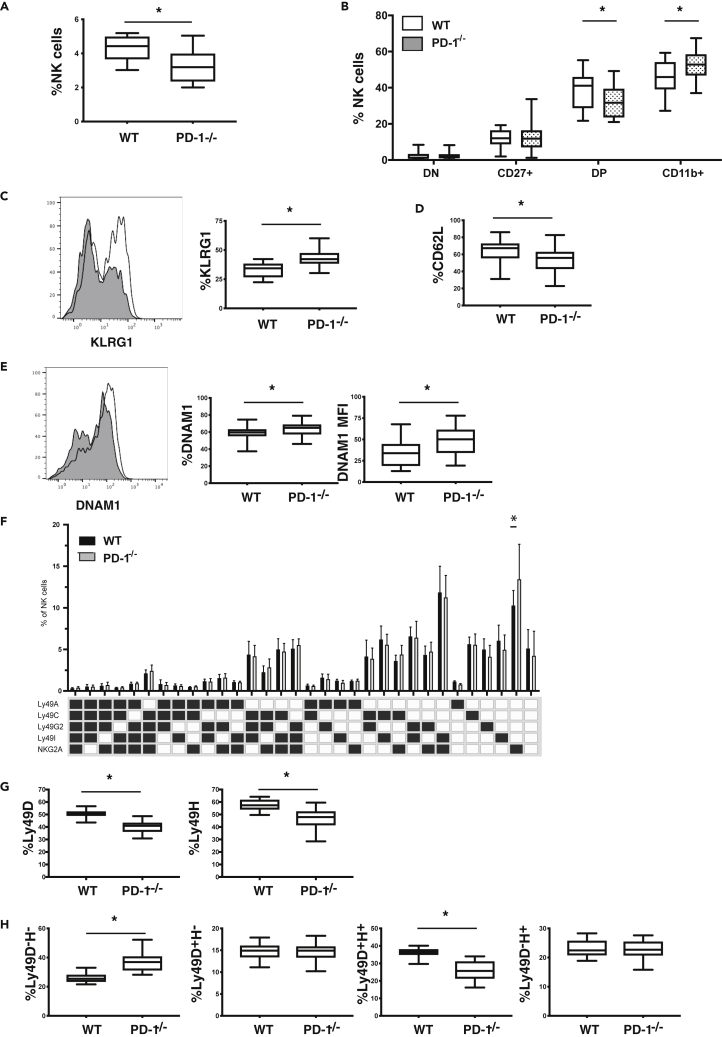
Phenotype of NK cells from WT and *PD-1*^*−/−*^ mice (A) Frequency of NK cells in the spleens of WT and *PD-1*^−/−^ mice (∗p<0.01 Mann-Whitney test, n = 18–20 mice/group data represent mean ± SD). (B) Expression of CD11b and CD27 on NK cells from WT (open boxplots) and *PD-1*^−/−^ (shaded boxplots) mice (∗p<0.01 Mann-Whitney test, n = 18–20 mice). (C) Expression of KLRG1 on NK cells from WT (shaded plot) and *PD-1*^*−/−*^ (open plot ) mice (∗p<0.01 Mann-Whitney test, n = 18–20 mice/group data represent mean ± SD). (D) Expression of CD62L on NK cells from WT and *PD-1*^*−/−*^ mice. (E) Expression of DNAM-1 on NK cells from WT (shaded plot) and *PD-1*^*−/−*^ (open plot) mice, bar graphs represent percent expressing cells and the mean fluorescent intensity of expression (∗p<0.01 Mann-Whitney test, n = 18–20 mice/group data represent mean ± SD). (F) Expression of inhibitory Ly49 molecules and NKG2A on NK cells from WT (open bars) and *PD-1*^*−/−*^ (shaded bars) mice (∗p<0.01 Mann Whitney n = 18–20 mice/group data represent mean ± SD). (G) Expression of activating Ly49 molecules on NK cells from WT and *PD-1*^*−/−*^ mice. (∗p<0.01 Mann-Whitney test, n = 18–20 mice/group data represent mean ± SD). (H) Expression of Ly49D and Ly49H populations on NK cells from WT and *PD-1*^*−/−*^ mice (∗p<0.01 Mann-Whitney test, n = 18–20 mice/group data represent mean ± SD).

Although PD-1 plays an important role in the development of ILCs (Yu et al., 2016), no studies to date have examined the phenotype of NK cells from PD-1-deficient mice. Furthermore, lack of PD-1 has been shown to affect T and B cell development, as well as their maturation (Ahn et al., 2018; Good-Jacobson et al., 2010; Nishimura et al., 1998; Odorizzi et al., 2015). On examination of the frequency of NK cells in wild-type C57Bl/6 (WT) and *PD-1*^−/−^ mice, we first observed a reduction of the frequency of NK cells in the spleens of *PD-1*^−/−^ mice (Figure 1A). When comparing the maturation status of NK cells between WT and *PD-1*^−/−^ mice (Hayakawa and Smyth, 2006), NK cells from *PD-1*^−/−^ mice exhibited an increase in frequency of mature phenotype (CD11b^+^CD27^−^) NK cells (Figure 1B). This appears to take place at the expense of CD11b^+^CD27^+^ NK cells as this subset was reduced in *PD-1*-deficient mice whereas the size of CD11b^−^CD27^+^ NK cell populations was not affected (Figure 1B). In line with this more mature phenotype, the frequency of the KLRG1^+^ NK cell subset (Huntington et al., 2007) was also increased in *PD-1-*deficient mice compared to WT mice (Figure 1C). In addition, the frequency of CD62L, which is important for NK cell migration (Persson and Chambers, 2011), was reduced in NK cells derived from *PD-1*-deficient mice (Figure 1D).

It has recently been shown that PD-1 affects DNAM-1 expression on CD8 T cells (Wang et al., 2018). We observed an increased frequency of DNAM-1^high^ NK cells and increased expression levels of DNAM-1 in *PD-1-*deficient mice compared to WT mice (Figure 1E). This confirms that PD-1 can modulate DNAM-1 expression not only on CD8 T cells but also on NK cells.

We further analyzed expression of inhibitory receptors on NK cells (Oberg et al., 2004), and compared the repertoire of inhibitory molecules on NK cells from WT and *PD-1*-deficient mice. We did not find any major differences in Ly49 receptor and NKG2A expression between these mice, apart from an increase in the NKG2A^single^ population on NK cells from *PD-1*^−/−^ mice (Figure 1F). The frequency of the activating Ly49D and Ly49H molecules was reduced in *PD-1*^−/−^ mice, and this appeared to be because of a reduction in the frequency of the Ly49D^+^Ly49H^+^ NK cell population (Figures 1G and 1H). The expression levels of other activating receptors including, for example, NKG2D and CD244, were not significantly different between WT and *PD-1*-deficient mice (Figure S1A and S1B).

Lack of PD-1 has been associated with the accumulation of exhausted T cells (Odorizzi et al., 2015). In addition, LAG3, CD39, and TIGIT can be used as markers for T cell exhaustion (Odorizzi et al., 2015). Comparing NK cells from WT and *PD-1*^−/−^ mice, we observed only small changes in the frequencies of CD39^+^ NK cells and LAG3^+^ NK cells in *PD-1*^−/−^ mice (Figures S1C and S1D). Surface expression of PD-L1, the ligand for PD-1, was similarly not significantly different between the two mouse strains (Figure S1H). In addition, we did not observe any difference in the expression of GITR, CXCR3 nor CXCR4 (Figures S1E–S1G).

To evaluate if the phenotypic changes that we observed on *PD-1-*deficient NK cells might be because of perturbations caused by T cells lacking PD-1 (Terme et al., 2011), we compared NK cells from *PD-1xRAG1*^−/−^ and *RAG1*^−/−^ mice because these mice lack both T and B cells. Similarly to T and B cell-competent mice, NK cell maturation was still skewed in *PD-1xRAG1*^−/−^ mice with increased frequencies of CD11b^+^CD27^−^ and KLRG1^+^ NK cells compared to *RAG1*^−/−^ mice (Figures S2A and S2B). However, we no longer observed any significant difference in the frequency of CD62L^+^ NK cells between *RAG1*^−/−^ and *PD-1xRAG1*^−/−^ mice (Figure S2C). DNAM-1 expression levels were still increased on NK cells from *PD-1xRAG1*^−/−^ mice but unlike in T and B cell-competent mice, the frequency of CD39-expressing NK cells was increased in *PD-1xRAG1*^−/−^ mice (Figures S2D and S2E). In contrast to *PD-1*^*−/−*^ mice, analysis of the expression levels of inhibitory receptors no longer revealed any difference in frequency of the NKG2A^single^ NK cell population between *RAG1*^−/−^ and *PD-1xRAG1*^−/−^ mice (Figure S2F).

Although the frequency of Ly49D^+^ NK cells was reduced in *PD-1xRAG1*^−/−^ mice, there was no difference in Ly49H expression between *RAG1*^−/−^ and *PD-1xRAG1*^−/−^ mice. The reduction in the Ly49D population appeared to be mostly in the Ly49D^+^Ly49H^−^ subset but not in the Ly49D^+^Ly49H^+^ population (Figures S2G and S2H). In summary, we observed in mice lacking PD-1 increased NK cell maturation combined with higher DNAM-1, KLRG1 expression and reduced Ly49D expression.

### Elimination of MHC-I-deficient cells is impaired in *PD-1*^−/−^ mice

**Figure 2:**
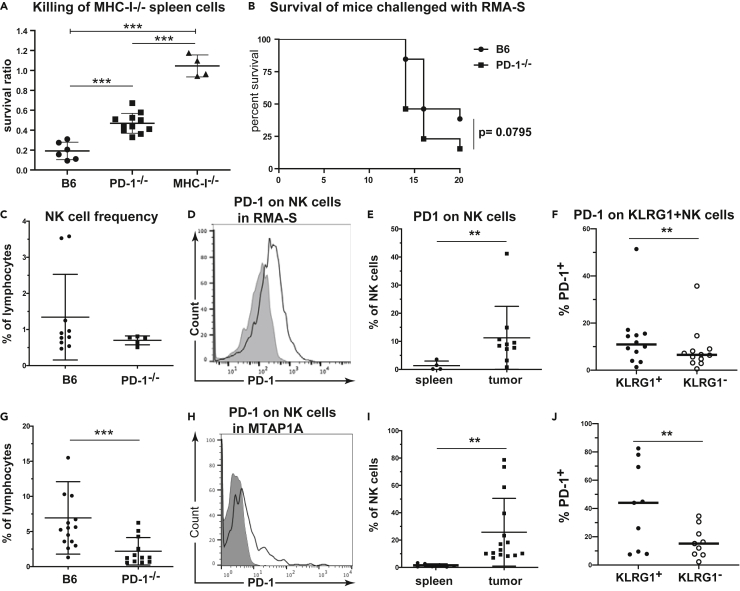
*PD-1-*deficient mice exhibit poor rejection of MHC-I-deficient cells (A) WT and *MHC-I*^−/−^ splenocytes labeled with CFSE were injected and the rejection ratio measured in WT (closed circle), *PD-1*^−/−^ (closed square) and *MHC-I*^−/−^ mice (closed triangle) ∗∗∗p<0.001 (ANOVA three separate experiments total of 6–8 mice/group data represent mean ± SD). (B) Rejection of RMA-S cells injected s.c. WT (closed circle) and *PD-1*^−/−^mice (closed square) were given an LD_50_ dose of RMA-S cells (10^5^ cells) and the survival rate of mice was measured (survival measured using log-rank test, three separate experiments with n= 15–16 mice). (C) Percent intratumoral NK cells amongst the lymphocyte population in WT mice (closed circle) or *PD-1*^−/−^mice (closed square) receiving RMA-S. (D and E) Expression of PD-1 on intratumoral NK cells in RMA-S treated mice compared to expression on splenocytes. Shaded background is based on the staining of PD-1 in *PD-1*^*−/−*^ mice (∗∗p<0.001 Mann-Whitney test, data represent mean ± SD). (F) Expression of PD-1 on intratumoral KLRG1^+^ (closed circle) and KLRG1^-^ (open circle) NK cell populations (p<0.05 paired t-test, data represent mean ± SD). (G) Frequency of intratumoral NK cells amongst lymphocytes in mice receiving MTAP1A, WT mice (closed circle) or *PD-1*^−/−^mice (closed square) (∗∗p<0.01 Mann-Whitney test, data represent mean ± SD). (H and I) Expression of PD-1 on intratumoral NK cells in MTAP1A treated mice compared to expression on splenocytes. Shaded background is based on the staining of PD-1 in *PD-1*^*−/−*^ mice (∗∗p<0.01 Mann-Whitney test, n = 12–15 mice, data represent mean ± SD). (J) Expression of PD-1 on intratumoral KLRG1^+^ (closed circle) and KLRG1^-^ (open circle) NK cell populations (∗∗p<0.01 paired t-test n = 8 data represent mean ± SD). Gating strategy for the NK cells is shown in Figure S4.

Chronic loss of PD-1 could potentially affect not only the phenotype of NK cells as outlined above, but also their function. The recognition and elimination of cells expressing reduced MHC-I levels is a hallmark of NK cell function and education (Brodin et al., 2009; Fernandez et al., 2005; Kim et al., 2005). We therefore examined the ability of *PD-1*-deficient and WT mice to eradicate MHC-I^neg^ spleen cells. In this competitive *in vivo* elimination assay, MHC-I^neg^ and MHC-I^pos^ spleen cells are differentially labeled, and co-administered *i.v.* (Wagner et al., 2016). Two days after injection, mice are sacrificed, and target cells are detected by flow cytometry in the spleen. The ratio between MHC-I ^neg^ and MHC-I^+^ target cells is given as the survival ratio of MHC-I^neg^ cells, as MHC-I^pos^ spleen cells will not be killed by syngeneic NK cells. We observed a significant reduction in the ability of *PD-1*^−/−^ mice to eliminate MHC-I^neg^ splenocytes compared to WT mice (Figure 2A). However, this impairment was not at the level seen in MHC-I^−/−^ mice.

It has been previously demonstrated that anti-PD-1 treatment increases NK cell elimination of MHC-I^neg^ PD-L1^+^ tumors (Hsu et al., 2018). To assess the tumor killing capacity of *PD-1*^−/−^ NK cells, mice were injected with an LD_50_ dose of TAP-deficient PD-L1^low^ RMA-S lymphoma cells. Killing of RMA-S is strongly dependent on NK cells, not T cells (Kärre et al., 1986). Although the survival rate of WT mice was 45% (6/13 mice), only 23% (3/13 mice) of *PD-1*-deficient mice survived (Figure 2B). Comparison of tumor infiltrating NK cells from WT and *PD-1*^−/−^ mice revealed a reduced frequency of tumor infiltration in *PD-1*-deficient mice (Figure 2C). PD-1 was heterogeneously expressed on NK cells infiltrating RMA-S in WT mice (Figure 2D), whereas splenic NK cells from the same mice exhibited little or no PD-1 expression (Figure 2E). PD-1 expression was higher on KLRG1^+^ NK cells than KLRG1^-^ NK cells (Figure 2F). Although these findings are similar to previous studies, the frequency of PD-1 expression on NK cells from our study were significantly lower (Hsu et al., 2018).

Next, we examined tumor infiltrating NK cells from the MHC-I^low^ tumor cell line MTAP1A, which is a fibrosarcoma generated from the skin of a *Tap1*-deficient mouse (Chambers et al., 2007). MTAP1A has low expression of PD-L1, and does not express PD-L2 (Figure S3). Here, again, we found reduced infiltration of NK cells in *PD-1*^−/−^ mice but increased expression of PD-1 on tumor-infiltrating NK cells in WT mice compared to splenic NK cells (Figures 2G–2I and S4).

In addition, PD-1^+^ tumor-infiltrating NK cells also displayed increased expression of KLRG1 compared to PD-1^neg^ NK cells (Figures 2F and 2J). This was observed for tumor-infiltrating NK cells in both RMA-S and MTAP1A, and suggested that PD-1-expressing NK cells might have a more mature phenotype.

### Single cell RNA-seq reveals tissue-specific transcriptional imprinting of tumor infiltrating NK cells

**Figure 3:**
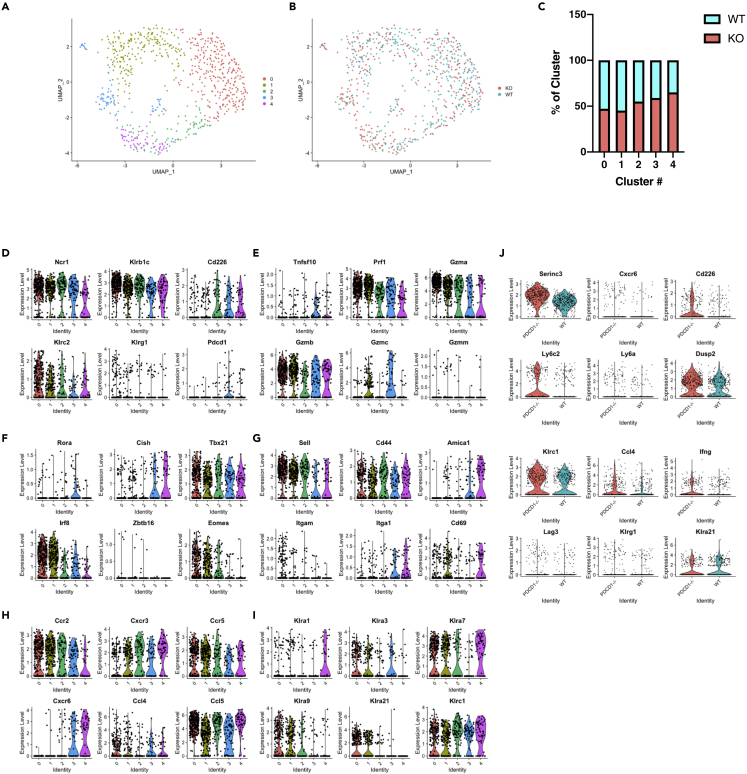
Single cell analysis of intratumoral NK cells (A and B) UMAP projection of 746 tumor-infiltrating NK cells (371 WT cells, 375 PD-1^−/−^ cells). (C) Percentage of each cluster derived from either WT or *PD-1*^*−/−*^ NK cells. (D–I) Violin plots for several genes enriched across various clusters. (J) Violin plots depicting expression of several genes differentially expressed significantly between WT and *PD-1*^*−/−*^ NK cells. The gating strategy for the sorting of NK cells is shown in Figure S5A.

Although it has been suggested that NK cells may express PD-1 through trogocytosis and because we observed differences in the phenotype of NK cells from WT and *PD-1*^−/−^ mice, we performed single cell RNA-sequencing (scRNA-SEQ) using the SMART-SEQ2 platform (Picelli et al., 2013) on tumor-infiltrating NK cells from mice inoculated with the MTAP1A tumor. We chose MTAP1A over RMA-S because this tumor model gave consistently higher frequency of PD-1-expressing NK cells. SMART-SEQ2 libraries of sorted NK cells were generated from pooled tumors from either WT or PD-1-deficient mice (Figures S5A and S5B). These libraries were filtered and a combined analysis was performed using Seurat v3 (Butler et al., 2018; Stuart et al., 2019) for a total of 371 WT and 375 *PD-1*^−/−^ NK cells after quality control (Figure S5C). Outliers expressing very few or too many genes were omitted, as were cells with a high frequency of apoptotic genes. Cells were clustered and projected using UMAP, which delineated five clusters with both WT and *PD-1*^−/−^ NK cells found in all clusters although *PD-1-*deficient cells were over-represented in clusters 3 and 4 (Figures 3A–3C). Differentially expressed (DE) genes were deciphered between all clusters and the top 10 genes per cluster shown by heatmap (Figure S6A). Selected genes were plotted using the Violin plot function revealing significantly over-expressed genes in each cluster. Within clusters 3 and 4, we could detect *Pdcd1* (PD-1) transcripts in both WT and *PD-1*^−/−^ NK cell populations, suggesting an active upregulation of *Pdcd1* at the transcriptional level (Figure 3D). Detection of *Pdcd1* transcript in *PD-1*^−/−^ mice reflects that these mice do not have a complete gene defect but rather a deletion spanning exon 3 and exon 4 of the P*dcd1* gene that prevents protein expression (Nishimura et al., 1998). Our analysis highlighted the heterogeneity of *in vivo* NK responses with distinct patterns of *Prf1*, *Gzma*, *Gzmb*, and *Gzmc* expression (Figure 3E).

Clusters 3 and 4 were defined by a paucity of *Eomes* and *Irf8* whilst being enriched for expression of *Tnfsf10* (TRAIL), *Cxcr6* and *Itga1* (CD49a) (Figures 3E–3G). These clusters also displayed greater expression of *Amica1*, *Ly6a*, *Il7r*, and *Il21r* and lower levels of *Sell* (CD62L) (Figures 3G and S6B). Cells found in cluster 3 also had significantly enhanced *Lag3* levels suggesting that this population may potentially harbor exhausted NK cells (Figure S6B). Taken together, these findings indicate that clusters 3 and 4 might represent a more mature/exhausted population and/or a tissue-resident-like subset of NK cells. Finally, we observed differential expression of transcripts for the inhibitory Ly49 genes *Klra1*, *Klra3*, *Klra7*, and *Klra9* amongst the different clusters. In particular, Klra3 (Ly49C) seemed to be present in cluster 3 but *Klra1* (Ly49A), *Klra7* (Ly49G2) and *Klra9 (Ly49I)* seemed to be under-represented in the same cluster (Figure 3I). This indicated that different Ly49 subsets of NK cells are present within the tumor microenvironment (TME) (Figure 3I) which may be explained by the observation that inhibitory Ly49 receptors specific for cognate MHC-I molecules were more likely to express PD-1 (Hsu et al., 2018).

Comparison of all WT with all *PD-1*^−/−^ NK cells independently of cluster identity determined a total of 54 genes that were significantly altered between these two NK cell populations (Figures 3J, S6C,Tables S1 and S2). Amongst those over-represented in *PD-1*^−/−^ NK cells were transcripts for *Cd226* (encoding for DNAM-1), *Klrc1* (NKG2A) and *Klrg1*, which is in line with our flow cytometry data on spleen NK cells. Furthermore, we found that *PD-1*-deficient NK cells had altered levels of expression for *Cxcr6* and select *Ly6* genes, suggesting that NK cells from *PD-1*^−/−^ mice had a more tissue-resident phenotype. *Ifng* and *Ccl4* transcripts were also more abundant in NK cells from *PD-1*^−/−^ mice indicating an influence of PD-1 on *in vivo* NK cell responses (Figure 3J). We also found that the intracellular levels of IFNγ in IL-12/15/18 cytokine-stimulated NK cells were increased in *PD-1*^−/−^ NK cells compared to NK cells from WT mice (Figure S6D), confirming that chronic lack of PD-1 might predispose NK cells to increased IFNγ expression.

### PD-1 expression is associated with the expression of CXCR6 in NK cells

**Figure 4:**
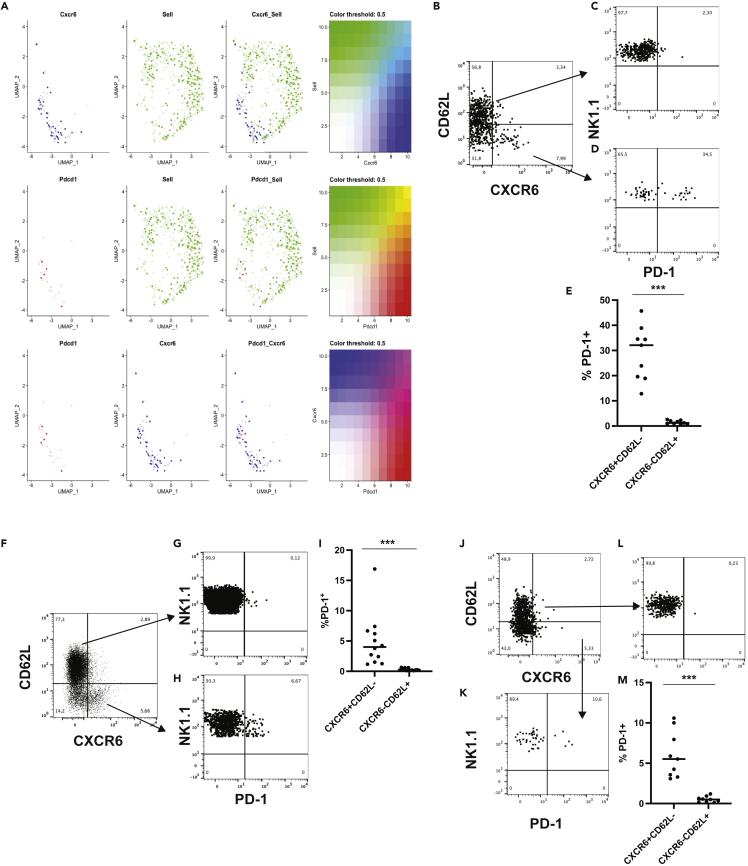
PD-1 expression on NK cells is primarily expressed on the CXCR6^+^ population of NK cells (A) UMAP projection of CD62L, CXCR6 and PD1 on the different clusters. CD62L and CXCR6 RNA expression was observed in different clusters (Upper panel). RNA expression of PD-1 and CD62L did not cluster together (Middle panel) but PD-1 and CXCR6 clustered together (Lower panel). (B) Expression of CD62L and CXCR6 on tumor infiltrating NK cells in MTAP1A. (C) Expression of PD-1 on CD62L^+^CXCR6^-^ NK cells. (D) Expression of PD-1 PD-1 on CD62L^−^CXCR6^+^ NK cells. (E) Comparison of PD-1 expression on CD62L^−^CXCR6^+^ and CD62L^+^CXCR6^-^ NK cells (∗p<0.001 Mann Whitney test n = 9 mice from three separate experiments, data represent mean ± SD). (F) Expression of CD62L and CXCR6 on spleen NK cells from RAG^−/−^ mice. (G) Expression of PD-1 on CD62L^+^CXCR6^-^ NK cells. (H) Expression of PD-1 PD-1 on CD62L^−^CXCR6^+^ NK cells. (I) Comparison of PD-1 expression on CD62L^−^CXCR6^+^ and CD62L^+^CXCR6^-^ NK cells (∗p<0.05 Mann Whitney test n = 12 mice, data represent mean ± SD). (J) Expression of CD62L and CXCR6 on tumor infiltrating NK cells in MTAP1A from *RAG*^*−/−*^ mice. (K) Expression of PD-1 on CD62L+ tumor infiltrating NK cells in MTAP1A from *RAG*^*−/−*^ mice. (L) Expression of PD-1 on CD62L^−^CXCR6^+^ tumor infiltrating NK cells in MTAP1A from *RAG*^*−/−*^ mice. (M) Comparison of PD-1 expression on CD62L^−^CXCR6^+^ and CD62L^+^CXCR6^-^ NK cells (∗p<0.001 Mann Whitney test n = 9 mice from three separate experiments,data represent mean ± SD).

To further demonstrate that a specific NK cell subsets could express PD-1, we visualized the distribution of RNA transcripts for CD62L, CXCR6, and PD-1 using blended Seurat FeaturePlots. CXCR6 did not appear to cluster together with CD62L, and similarly neither did PD-1. However, expression of CXCR6 and PD-1 was mapped together in the same clusters (Figure 4A).

Because the RNA expression profile of NK cells expressing PD-1 and CXCR6 suggested that they may be connected, we compared the expression of PD-1 on CXCR6 and CD62L subsets of tumor infiltrating NK cells in the MTAP1A tumor model by flow cytometry. Although the frequency of PD-1-expressing tumor infiltrating CD62L^+^CXCR6^-^ NK cells was low (Figures 4C, 4E, and S7, 1.5%±0.7 n = 9), PD-1 expression was associated with the CD62L^−^CXCR6^+^ NK cells, with approximately 25% of tumor infiltrating CXCR6^+^ NK cells expressing PD-1 (Figures 4D and 4E, 29% ± 11 n = 9). The expression of PD-1 on CD62L^−^CXCR6^-^ NK cells was higher than that on CD62L^+^CXCR6^-^ NK cells but lower than that observed on CD62L^−^CXCR6^+^ NK cells (5.3 ± 1.6% n = 9).

When we examined the splenic NK cells from RAG1-deficient mice, we could still find an association between PD-1 and CXCR6^+^ NK cells (Figures 4F–4I and S8). However, when RAG1-deficient mice were inoculated with MTAP1A, we found few CXCR6^+^ intratumoral NK cells (Figure 4J), however, even in the RAG1-deficient mice PD-1 expression was associated with the CD62L^−^CXCR6^+^ population of NK cells (Figures 4J–4M).

When we examined intratumoral NK cells from mice inoculated with RMA-S, we found that these cells also had reduced frequency of CXCR6 when compared to intratumoral NK cells from mice inoculated with MTAP1A (Figure S9A). However, PD-1 expression on NK cells was still associated with the CXCR6^+^ NK cells (Figure S9B).

### PD-1 can form *cis* interactions with PD-L1 on NK cells

**Figure 5:**
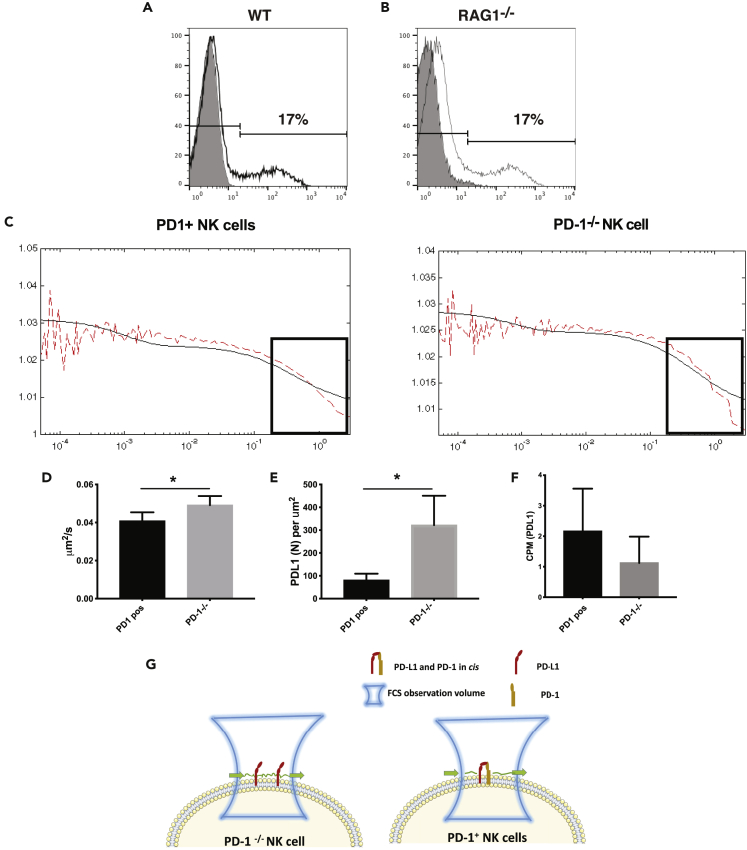
Movement of PD-L1 in WT and *PD-1*^*−/−*^ NK cells (A) Isolated splenic NK cells stimulated with IL-12, IL-15 and IL-18 can express PD-1, A, wild-type B6 mice, B, *RAG1*^*−/−*^ mice. Shaded background is based on the anti-PD-1 antibody staining of in the respective PD-1 deficient mice. (C) Representative FCS auto correlation curves of PD-L1 on PD-1 positive (*right panel*) and *PD-1*^*−/−*^ NK cells (*left panel*), decline part of the curve indicates the rate of diffusion on cell membrane. FCS readouts of PD-L1 molecule on PD-1 positive and negative NK cells. (D) The diffusion rate of PD-L1, E, the density of PD-L1 and F, the counts per molecule (CPM) of PD-L1 were measured on individual PD-1^+^ NK cells from *RAG1*^−/−^ mice and NK cells from *PD-1xRAG1*^−/−^ mice. D, The diffusion rate of PD-L1 is faster in the absence of PD-1 (∗∗∗p<0.001 Mann-Whitney test n = 10–12 NK cells/group, data represent mean ± SD). (E and F) The density of PD-L1 was higher on *PD-1xRAG1*^−/−^ NK cells (∗∗∗p<0.001 Mann-Whitney test n = 10–12 NK cells/group, data represent mean ± SD), whereas in F, the CPM, indicates the size of the cluster measured based on the brightness or number of molecules per entity, PD-L1 clusters was higher when PD-1 was present. (G) Model for PD-L1 movement in the membrane in the presence and absence of PD-1.

Because the expression profile of NK cells expressing PD-1 and CXCR6 suggested that they may be connected, we stimulated enriched NK cells from WT mice with a combination of IL-12/15/18 cytokines for 96 h, which has previously been show to induce memory NK cells (Cooper et al., 2009) as well as CXCR6 on the surface of NK cells (Hydes et al., 2018). This cytokine stimulation resulted in approximately 10% of WT NK cells expressing PD-1 (Figures 5A and S10). Similar patterns of staining were seen in cytokine-stimulated NK cells from *RAG1*^−/−^ mice (Figure 5B), which ruled out that expression of PD-1 might be on a T cell subset with low CD3 expression, that T cells could induce PD-1 on NK cells or that PD-1 expression was because of trogocytosis from T cells. Because PD-1 and PD-L1 have recently been shown to form *cis*-interactions in artificial lipid structures and in antigen-presenting cells (APCs) (Zhao et al., 2018), we investigated whether PD-L1 on non-tumor cells could interact with PD-1 on NK cells using these IL-12/15/18 stimulated NK cells. It has been shown previously that inhibitory MHC-I-binding molecules on NK cells could form *cis*-interactions with their ligands (Bagawath-Singh et al., 2016; Chalifour et al., 2009). We therefore assessed whether the movement of PD-L1 was restricted in the presence of PD-1 and determined PD-L1 diffusion on the membranes of NK cells lacking PD-1 compared to WT NK cells using fluorescence correlation spectroscopy (FCS). This method detects diffusion of molecules and it has previously been used to measure the diffusion of receptors in the membrane of NK cells (Bagawath-Singh et al., 2016; Guia et al., 2011). A series of autocorrelation curves were generated and fitted to the 2D diffusion FCS curve fitting equation. Representative autocorrelation curves with 2D curve fit are shown in Figure 5C. Of interest, PD-L1 diffused significantly faster on the membrane of NK cells lacking PD-1 compared to PD-1^+^ NK cells from WT mice (Figures 5C and 5D). Furthermore, we observed a trend for high levels of PD-L1 molecules per μm^2^ on the surface of NK cells lacking PD-1 (Figure 5E). Because molecule crowding factor is ruled out on PD-1^+^ NK cells, the slow diffusion of PD-L1 molecules on cell membranes can be due to specific interactions or clustering. To investigate whether PD-1 and PD-L1 form clusters on the surface of NK cells, the brightness of PD-L1 was quantified, which is measured in terms of counts per molecule diffusing within the observation volume. We observed a tendency toward larger clusters, as the brightness of PD-L1 on PD-1 positive NK cells was higher compared to *PD-1*^−/−^ NK cells (Figure 5F). These data suggest that PD-L1 on PD-1^+^ NK cells clusters with PD-1, indicating *cis* interactions on the membrane of NK cells. In conclusion, PD-L1 diffuses faster without any hinders on *PD-1*^−/−^ NK cells whereas in presence of PD-1 on cell membrane PD-L1 diffuses slower, which suggests that PD-L1 might be clustering in *cis* with PD-1 on the cell membrane (Figure 5G).

**Figure 6:**
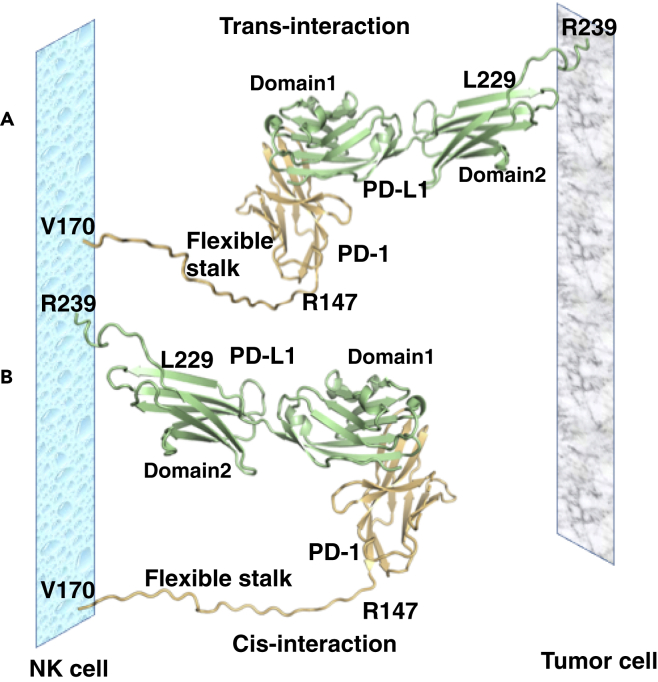
Molecular Modeling of PD-1-PD-L1 *cis*-interaction The 24 amino residues long stalk region of PD-1 is long and flexible enough to allow for both *trans*- and *cis*-interaction between PD-1 and PD-L1. (A) *Trans*-interaction between PD-L1 on tumor cells and PD-1 on NK cells. (B) *Cis*-interaction between PD-1 and PD-L1 on NK cells. The interactions and mode of binding between the N-terminal part of PD-L1 and PD-1 could be highly similar, as found in the crystal structure of the human PD-1/PD-L1 complex (Zak et al., 2015). The stalk-region of PD-1 (residues R147-V170) was modeled in an arbitrary extended conformation to show that its length is sufficient to allow for *cis*-interaction with the N-terminal domain of PD-L1.

Three-dimensional molecular models of the full-length extracellular domains of PD-1 and PD-L1 reveal that their structural features easily allow for the formation of *cis*-interactions. Indeed, a model of the stalk region of PD-1 (comprising the stretch of residues R147-V170) in extended conformation demonstrates that its length is sufficient to allow both *cis*- and *trans-*interactions with the N-terminal domain of PD-L1 (Figure 6). Our molecular models thus suggest a binding in which PD-1 “tip-toes” to reach PD-L1 with an extended stalk, while keeping the same PD-1/PD-L1 “cheek-to-cheek” interface found in previous crystal structures (Figure 6).

## Discussion

Expression of PD-1 on NK cells has been observed in many human and mouse studies (Alvarez et al., 2020; Beldi-Ferchiou et al., 2016; Benson et al., 2010; Guo et al., 2016; Hsu et al., 2018; MacFarlane et al., 2014; Norris et al., 2012; Terme et al., 2011). However, some recent studies suggested that NK cells do not express PD-1 and expression may because of artifact of flow cytometry staining or through interactions with PD-L1 and the NK cells acquiring PD-1 via trogocytosis (Hasim et al., 2022; Judge et al., 2020). However, in the present study, we could find transcript and surface expression of PD-1 in tumor infiltrating NK cells which was associated with the expression of CXCR6. This association between PD-1 and CXCR6 was also observed on IL-12/15/18 stimulation of NK cells in culture. Furthermore, PD-1 was induced on tumor-infiltrating NK cells even though the tumors themselves expressed little or no PD-L1. NK cells from mice lacking PD-1 displayed phenotypic differences compared to NK cells from WT mice, suggesting that background low levels of PD-1 might still play a role in NK cell homeostasis or in NK cell development. In particular, NK cells from *PD-1-*deficient mice exhibited increased maturation as well as increase in expression of DNAM-1.

We found that *PD-1*-deficient mice were poor at rejecting MHC-I^neg^ cells and had low NK cell infiltration into tumors expressing low levels of MHC-I *in vivo*. In part, this might be because of the increased maturity of NK cells in *PD-1*-deficient mice, but the reduced frequency of tumor infiltrating NK cells could also be because of (1) reduced CD62L found on the *PD-1*^*−/−*^ NK cells or (2) reduced survival once these NK cells encounter tumor cells.

A recent study reported that PD-1 antibody blockade increases the immune response of NK cells both in an MHC-I^neg^ and MHC-I^pos^ tumor setting (Hsu et al., 2018), supporting the notion that NK cells participate in the clinical benefit of PD-1/PD-L1 antibody therapy. However, in this study Hsu et al., also demonstrated that RMA-S expressing PD-L1 grew out faster and was more lethal than RMA-S that did not express PD-L1 (Hsu et al., 2018) which further suggested that there was a role of PD-1 on NK cells in eliminating this tumor. Our data has some differences to their data which may be because of the fact that our tumors had low expression of PD-L1 and that we used mice lacking PD-1. This chronic loss of PD-1 leads to other changes in NK cells such as increased maturation that could have altered the ability of these NK cells to kill tumors or to migrate to tumors because of changes in the environment because of, e.g., cytokines that are released.

Our data, that the chronic lack of PD-1 leads to an impairment of anti-tumor and missing self-responses of NK cells, are reminiscent of previous studies showing that short-term blockade of inhibitory NK cell receptors increases their anti-tumor function, whereas longer blockade leads to a reduction of the function (Carlsten et al., 2016; Wagner et al., 2016). In the case of inhibitory receptors that recognize MHC-I, these observations were explained by the ability of NK cells to retune their responsiveness to the new integrated input of activating and inhibitory signals during the chronic blockade of inhibitory signals. Although we could not detect an education defect *per se* in the *PD-1*^*−/−*^ mice, we observed decreased cytotoxicity toward MHC^neg^ spleen cells which represent missing self targets. In recent years, the important roles of non-MHC-I molecules in NK cell education have been highlighted (He and Tian, 2017), and our observations pertaining to NK cell functionality in *PD-1*^*−/−*^ mice could indicate that this inhibitory receptor may have a minor role in setting the threshold of NK cell responsiveness in steady state.

It is unclear if the increased NK cell maturation that we observed in the *PD-1*^*−/−*^ mice is a direct effect on NK cells because we observe very little or no PD-1 on NK cells in circulation. It should be noted though that, lack of PD-1 on dendritic cells leads to increased IL-12 and TNF production by dendritic cells (Yao et al., 2009). Thus, lack of PD-1 on DCs could indirectly affect NK cell maturation. Furthermore, absence of PD-1 on T and B cells could affect NK cells indirectly as well (Chambers, 2009; Kerdiles et al., 2013). However, even in the *PD-1xRAG1*^−/−^ mice, we still had more mature NK cells and increased expression of DNAM-1 suggesting that the *PD-1*^−/−^ T and B cells had little effect on the NK cell phenotype. *PD-1*^−/−^ NK cells stimulated with IL-12/15/18 had increased numbers of IFNγ-producing cells suggesting that increased IL-12 from accessory cells in *PD-1*^−/−^ mice (Yao et al., 2009) might already prime NK cells to make more IFNγ. Chronic infection and IL-18 expression have previously been associated with higher expression of PD-1 on NK cells (Alvarez et al., 2010; Golden-Mason et al., 2008; Norris et al., 2012; Quatrini et al., 2018, 2021; Terme et al., 2011). Even though PD-1 expression on T cells has been associated with exhaustion, it may also be a marker for activation and that its expression controls T cells from being overly activated (Odorizzi et al., 2015; Schietinger et al., 2016). Thus, PD-1 expression on NK cells might play a similar role within the frame of NK cell activation (Hsu et al., 2018).

Recent studies have called into question whether PD-1 is actually expressed at all on NK cells (Hasim et al., 2022; Judge et al., 2020). Our results are in agreement with some of these findings, including the low surface of expression of PD-1 on NK cells under physiological conditions. A number of articles have detected transcript for PD-1 in NK cells that could be reduced with IL-2 stimulation (Judge et al., 2020) or controlled by pro-inflammatory cytokines and glucocorticoids (Quatrini et al., 2018, 2021). However, Hasim et al. did not detect PD-1 on NK cells stimulated with a variety of cytokines (Hasim et al., 2022). Because PD-1 is expressed on other tumor-infiltrating cells, there is still the possibility that PD-1 may be transferred by trogocytosis from surrounding cells to NK cells via SLAM receptors (Hasim et al., 2022; Judge et al., 2020). Because we observed a paucity of *PDCD1* transcript in the CD62L^+^ tumor infiltrating NK cells, we believe that PD-1 surface expression on these NK cells could be because of trogocytosis. However, because we see both the majority of the *PDCD1* transcript and surface expression of PD-1 on the CXCR6^+^ NK cells, it suggests that expression of PD-1 on these NK cells is not because of trogocytosis. Finally, Metzger et al. have also suggested that false positives can be obtained by anti-PD-1 antibodies binding to nuclear antigen in dying cells (Metzger et al., 2018). In our studies, we have compared our staining of WT NK cells with NK cells from *PD-1*^*−1*^^−^ mice and did not see non-specific binding using the anti-PD-1 antibody clone RMP1-14 or clone 29F.1A12. We did not use the J43 clone in our studies because we had previously found some non-specific binding with this clone. This suggested that, at least in our hands, our observed PD-1 expression was not due to cross-reactivity with another antigen.

NK cells play an important role in clearance of tumor cells, and impairment of NK cell functions results in an increased risk for the development of cancer. Both tumor-infiltrating NK cells (TINKs) and tumor-associated NK cells (TANKs) have been described (Bruno et al., 2014), but their function and expression profiles have yet to be defined. Our single cell gene expression data reveal that NK cells within the TME separate into five distinct clusters. Many DE genes of our intratumoral NK cells have been previously described in tissue-resident NK cells in different organs including liver, lung, lymph node, and placenta. The high expression of tissue residency markers in NK cells within the TME could indicate that these NK cells are tumor tissue-resident. Whether these NK cells infiltrate tumors (TINKs) to eliminate them, or whether they associate with tumor cells (TANKs) and facilitate pro-angiogenic properties, remains difficult to assess. In many tumors, TINKs exhibit a profoundly altered phenotype with defects in degranulation and IFNγ expression (Platonova et al., 2011). Our finding that PD-1^+^ NK cells within the TME co-express CXCR6 is interesting in light of a recent report suggesting that CXCR6 on cytotoxic T cells enables them to receive critical survival signals within the TME (Di Pilato et al., 2021). However, it is still unclear whether PD-1 on tissue resident-like NK cells is because of exhaustion and functional impairment, or if expression of PD-1 restricts NK cell activation and terminal maturation to prevent the exhausted phenotype, as has been suggested for T cells (Alvarez et al., 2020; Odorizzi et al., 2015).

Previous work has established the bidirectional signaling of PD-1 and PD-L1. *Cis* interactions between PD-1 and PD-L1 on antigen-presenting cells have been shown to decrease availability of PD-L1 for *trans* binding to PD-1 on T cells, and both *cis* and *trans* interactions are susceptible to antibody blockade (Zhao et al., 2018). In the current study, we have shown that *cis* interaction between PD-1 and PD-L1 and a potential sequestration of available PD-1 for *trans* signaling also occurs on NK cells. We show that in the absence of PD-1, the diffusion rate of PD-L1 is significantly increased, whereas the size of PD-L1 clusters is decreased, indicating that PD-L1 forms clusters with PD-1 on the same membrane, thus limiting the movement of PD-L1 and potentially also that of PD-1. This suggests that the levels of PD-L1 on NK cells can determine their response to PD-1 signaling imposed by PD-L1^+^ cells inside the TME. We further provide a model for how PD-1 and PD-L1 interact in *trans* and in *cis*, where the same amino acid residues are involved in these interactions.

The binary PD-1/PD-L1 complex was crystallized both for human PD-L1 and murine PD-1 (Lin et al., 2008) and for human PD-L1 and human PD-1 (Zak et al., 2015). In both cases, protein-protein binding occurs via “cheek to cheek interaction” of Ig domains of PD-1 and PD-L1, and this was almost identical in the two structures. We hypothesize that the long flexible stalk of PD-1 allows both *cis* and *trans*interaction, where PD-1 “tip-toes” to reach PD-L1 with an extended stalk, whereas keeping the same PD-1/PD-L1 “cheek-to-cheek” interface found in the crystal structures. The stalk region of PD-1 (residues R147-V170) was modeled in extended conformation to demonstrate that its length is sufficient to allow both *cis*- and *trans-*interaction with the N-terminal domain of PD-L1. High sequence homology between murine and human proteins (77% for PD-L1 and 64% for PD-1) and conservation of the residues forming intermolecular hydrogen bonds suggest that the *cis* and *trans*-interaction for the PD-1 and PD-L1 could be possible for the human cells as well. Indeed, *cis* binding of human PD-1 and human PD-L1 has recently been demonstrated (Zhao et al., 2018).

A recent study has shown that NK cells up-regulate PD-L1 in response to IFN-γ and that NK cells from AML patients show increased expression of PD-L1 (Dong et al., 2019). PD-L1^+^ cells in the TME negatively regulate PD-1^+^ effector cells, but at the same time, PD-L1 on T and NK cells might inhibit survival of PD-1^+^ APCs (Park et al., 2014). In addition to binding PD-1 in *cis*, PD-L1 can also bind to CD80 on the same membrane, which may repress both PD-1 and CTLA-4 signaling while favoring the CD28 axis (Zhao et al., 2019). These multi-facetted binding patterns in *trans* and *cis* may contribute to the fine-tuning of the immune response within the TME, and may be the cause for the differences observed when treating cancer patients with anti-PD-1 versus anti-PD-L1 blocking antibodies (Duan et al., 2019).

Since we find that the PD-1 tumor-infiltrating NK cells are expressed primarily in the CXCR6^+^ NK cell population, this may open up new possiblities to investigate the function of NK cells in the setting of PD-1/PD-L1 therapy. PD-1 blockade is effective in human tumors that have lost HLA-I expression or show low levels of mutational load, both factors necessary for a T cell-mediated response (Ansell, 2016; Ansell et al., 2015). Furthermore, even in tumors where the effect of PD-1 therapy is clearly T cell-mediated, the presence of innate immune cells such as DCs and NK cells within the TME were the strongest predictors or responsiveness (Barry et al., 2018). In these settings, NK cells may be enhanced to kill tumor cells directly, thus contributing to the antigen presentation by APCs, or by helping to recruit an adaptive immune response.

### Limitations of the study

Despite several recent publications showing PD-1 on NK cells in human tumors, there is still a controversial discussion whether NK cells themselves actually express PD-1. In our study, we show PD-1 expression on RNA and protein level, albeit in murine not human NK cells. Our study on tumor-infiltrating NK cells have been conducted in mice with a general deletion in the PDCD1 gene. This could potentially lead to changes in NK cell function and phenotype because of PD-1 deletion in other immune cells. We tried to address this concern by looking at NK cells from B cell and T cell deficient mice, and could show that the major effects of chronic absence of PD-1 are NK cell effects.

Another limitation of our study is the use of two tumor models that are known to be primarily targeted by NK cells, not T cells. How the observed effect will translate into a tumor setting with heterogeneous expression of MHC-I and contribution of T cell-mediated and NK cell-mediated cytotoxicity, needs to be addressed in future studies.

## STAR★Methods

### Key resources table

**Table:** 

REAGENT or RESOURCE	SOURCE	IDENTIFIER
**Antibodies**		

NK1.1 APC	Biolegend	Cat# 108710 RRID AB_313397
NK1.1 APC Cy7	Biolegend	Cat# 108724 RRID AB_830871
NKp46 BV421	Biolegend	Cat#137611 RRID AB_10915472
PD-1 PE	Biolegend	Cat# 135205 RRID AB_1877232
PD-1 APC	Biolegend	Cat# 135210 RRID AB_2159183
PD-1 APC	Biolegend	Cat# 114118 RRID AB_2566726
PD-1 Alexa 488	R&D systems	Cat# FAB7738G
PD-L1 Alexa 647	R&D systems	Cat# FAB9078R
NKG2A APC	BD	Cat# 564383 RRID AB_2738783
Ly49A Pacific Blue	Biolegend	Cat# 116810 RRID AB_572013
Ly49D FITC	Biolegend	Cat#138303 RRID AB_10588709
Ly49G2 FITC	eBioscience	Cat #11-5781-82 RRID AB_763604
Ly49H APC	eBioscience	Cat# 17-5886-82 RRID AB_10598809
Ly49I PE	eBioscience	Cat# 12-5895-82 RRID AB_446021
CD11b FITC	Biolegend	Cat# 101205 RRID AB_312788
CD127 BV650	Biolegend	Cat# 135043 RRID AB_2629681
CD90.2 APC Cy7	Biolegend	Cat# 140331 RRID AB_2894662
CD45.2 BV421	BD	Cat# 562895 RRID AB_2737873
GITR PE	Biolegend	Cat#126309 RRID AB_1089132
CD244 FITC	eBioscience	Cat# 11-2441-82 RRID AB_657875
TIGIT	eBioscience	CAT# 67-9501-82 RRID AB_2723713
CD39 PE	Biolegend	Cat# 143803 RRID AB_11219591
LAG3 PE	BD	Cat# 552380 RRID AB_394374
KLRG1 FITC	Biolegend	Cat# 138410 RRID AB_10643582
KLRG1 PerCp Cy5.5	Biolegend	Cat# 138418 RRID AB_2563015
CD226 Alexa 647	Biolegend	Cat# 128808 RRID AB_1227541
CD62L APC	Biolegend	Cat# 104412 RRID AB_313099
CD62L FITC	Biolegend	Cat# 104406 RRID AB_313093
GR1 PerCP Cy5.5	Biolegend	Cat# 108428 RRID AB_893558
GR1 Biotin	Biolegend	Cat# 108404 RRID AB_313369
CXCR3	eBioscience	Cat#126516 RRID AB_2245493
CXCR4	Biolegend	Cat# 146511 RRID AB_2562788
CXCR6 PE Cy7	Biolegend	Cat# 151118 RRID AB_2721669
CD274 PE	Biolegend	Cat# 155403 RRID AB_2728222
CD3 PerCP Cy5.5	Biolegend	Cat# 100327 RRID AB_893320
CD3 Biotin	Biolegend	Cat# 100303 RRID AB_312668
CD19 Biotin	Biolegend	Cat# 115503 RRID AB_313638
IFNγ APC	Biolegend	Cat# 505809 RRID AB_315403
CD27 PE	Biolegend	Cat# 124209 RRID AB_1236464
Streptavidin PerCp Cy5.5	Biolegend	Cat# 405231
Streptavidin BV650	Biolegend	Cat# 405214
Ly49C (4LO3311)	Gift	Susanne Lemieux
Anti-CD16/CD32	Mabtech	N/A

**Chemicals, peptides, and recombinant proteins**		

rmIL-12	Peprotech	Cat# 210-12
rmIL-15	Immunotools	Cat # 12340155
rmIL-18	MBL	Cat# B001-5
CFSE	ThermoFisher	Cat# C34554
Cell trace Violet	ThermoFisher	Cat# C34557
Live/Dead Fixable Aqua Dead Cell Stain	ThermoFisher	Cat# L34966
Fixable Viability Dye eFluor 780	ThermoFisher	Cat# 65-0865-14
RPMI	HyClone	Cat# 16750-084
FBS	Gibco	Cat# 10270-106
2-mercaptoethanol	Gibco	Cat# 31350-010
HEPES	HyClone	Cat# SH30237.01
L-glutamine	HyClone	Cat# SH30034.01
Sodium Pyruvate	HyClone	Cat# SH30239.01
4% Formaldehyde	ITW Reagents	Cat# 252931.1211
Intracellular staining	Biolegend	Cat# 421002

**Critical commercial assays**		

NK cell isolation kit	Miltenyi Biotech	Cat# 130-115-818

**Deposited data**		

Smart-Seq2 data	This study	GSE211488

**Experimental models: Cell lines**		

RMA-S	(Karre et al., 1986)	N/A
MTAP1A	(Chambers et al., 2007)	N/A

**Experimental models: Organisms/strains**		

C57Bl/6JRj	Janvier	N/A
PDCD1−/−	Riken	N/A
RAG1−/−	Jackson Laboratories	N/A
PDCD1−/−x RAG1−/−	(Smith et al., 2016)	N/A
H-2K^b^xH-2D^b−/-^	(Hoglund et al., 1998)	N/A

**Software and algorithms**		

GraphPad Prism 9	Graph Pad	N/A
FlowJo	Biolegend	N/A
MATLAB	Mathworks	N/A
Biorender	Biorender	https://biorender.com
Seurat 3.0	(Stuart et al., 2019)	https://satijalab.org/seurat/
Swiss Model	(Waterhouse et al., 2018)	https://swissmodel.expasy.org/

**Other**		

Protein Data base	https://www.rgsb.org	3BIK, 4ZQK

### Resource availability

#### Lead contact

Further information and requests for resources and reagents should be directed to and will be fulfilled by the lead contact, Benedict Chambers (benedict.chambers@ki.se).

#### Materials availability

This study did not generate new unique reagents.

### Experimental model and subject details

#### Mice

C57BL/6, *PDCD-1*(PD-1)^−/−^ (generously provided Dr. Tasuku Honjo, Kyoto University, Kyoto, Japan) (Nishimura et al., 1998), *RAG1*^−/−^(Mombaerts et al., 1992) and *PD-1*^−/−^x*RAG1*^−/−^ (PD-1xRAG1^−/−^) (Smith et al., 2016), *H-2K*^*b*^*xH-2D*^*b−/-*^(MHC-I^−/−^) (Hoglund et al., 1998) mice on the C57BL/6 background were housed under specific pathogen free conditions at the Department of Microbiology, Tumor and Cell Biology and Astrid Fagraeus Laboratories, Karolinska Institutet, Stockholm. All procedures were performed under both institutional and national guidelines (Ethical numbers from Stockholm County Council N147/15). Sex and aged match mice (8–12 week old) were used for all experiments. Mice were chosen randomly for control or treated groups.

#### Tumors

MHC-I-deficient lymphomas RMA-S (*TAP2*-deficient), and *TAP1*-deficient MCA fibrosarcoma (clone MTAP1A) have been previously described (Chambers et al., 2007; Kärre et al., 1986). Cells were thawed prior to use and grown in complete medium (RPMI; 10 mM HEPES, 2 × 10^−5^ M 2-ME, 10% FCS, 100 U/ml penicillin, 100 U/ml streptomycin). RMA-S cells were inoculated at the LD_50_ dose of 10^5^ s.c. in the flank of mice. MTAP1A was inoculated at a dose of 10^5^ cells/mouse. Since the sex of the tumors was unknown, male mice were used as recipients. Tumor growth was measured every two days and mice were sacrificed when the tumor reached 10^3^ mm.

### Method details

#### NK cell purification and culture

Single-cell suspension from spleens was depleted of erythrocytes, and NK cells were positively sorted by negative sorting using MACS separation, using NK cell isolation kit from Miltenyi Biotec (Miltenyi Biotec, Bergisch Gladbach, Germany). Cells were resuspended in complete medium (RPMI; 10 mM HEPES, 2 × 10^−5^ M 2-ME, 10% FCS, 100 U/ml penicillin, 100 U/ml streptomycin) with with 100 ng/mL mouse IL-12 (PeproTech), IL-15 (Immunotools) and 100 ng mouse IL-18 (MBL International, Woburn, MA, USA) for four days. For isolation of NK cell subsets, NK cells were isolated as above and then sorted on BD Influx (Becton Dickenson, CA, USA).

#### *In vivo* rejection assay

Splenocytes from B6 or *MHC-I*^*−/−*^ mice were labeled with 0.5 μM CFSE (target cells) or 0.5 μM CellTrace Violet (control cells; Thermo Fisher Scientific Life Sciences) for 10 min. Target and control cells were washed, then mixed and 1–3 × 10^6^ cells coinjected intravenously via the tail vein into B6, *PD-1*^*−/−*^ mice or *MHC-I*^*−/−*^ mice as controls for NK cell-mediated killing. The injection mix was analyzed by flow cytometry for reference. Two days later, the spleens were harvested and erythrocytes depleted, and the relative percentages of target and control cells were measured by flow cytometry (Oberg et al., 2004). Rejection was estimated as the relative survival of target or cells, calculated as: % remaining target cells of labeled cells/% target cells in inoculate or % remaining control cells of labeled cells/% control cells in inoculate.

#### Flow cytometry

Splenic and tumor NK cells were stained after single cell preparations were depleted of erythrocytes. Cells were stained as outlined in the various experiments and the antibodies used outlined above. For gating of PD-1 stainings, cells from *PD-1*^*−/−*^ mice were used as negative controls. For cultured NK cells, cells were incubated with monensin and Brefeldin A for four hours and stained for surface markers before being fixed in formaldhyde. Intracellular staining for IFNγ was performed using Biolegend’s intracellular staining kit. Flow cytometry was performed on CyAN ADP LX 9-colour flow cytometer (Beckman Coulter, Pasadena, CA) or LSRII (Becton Dickinson). Data were analyzed using FlowJo software (Tree Star Inc, OR).

#### Molecular modelling of cis- and trans-interactions between PD-1 and PD-L1

Three-dimensional molecular models of the full-length extracellular regions of murine or human PD-1/PD-L1 complexes (PD-L1 residues 19-239 and PD-1 residues 21-170) were created based on the crystal structure of the chimeric complex of human PD-L1 and murine PD-1 (pdb code3BIK) (Lin et al., 2008). To our knowledge, no crystal structure of murine PD-L1 has been determined yet, although several crystal structures of human PD-L1 are available (Chen et al., 2010; Lin et al., 2008).The crystal structure of human PD-L1 revealed that it consists of two Ig domains linked by a 10 residues-long stalk region. The sequence identity between murine and human PD-L1 is 77%, which means that their 3D structures may be very similar. Indeed, the model of murine PD-L1 created using SwissModel (Waterhouse et al., 2018) is very similar to human PD-L1. Replacement of human PD-L1 with its murine orthologue in the 3BIK structure allowed us to generate a full-length model of the murine PD-1/PD-L1 complex. Conversely, replacement of murine PD-1 with the human orthologue allowed us to create a three-dimensional model of the full-length human PD-1/PD-L1 complex. The stalk regions of PD-1 (residues 147-170) and PD-L1 (residues 229-239) were modelled in an arbitrary extended conformation using the program Coot (Emsley et al., 2010) followed by model regularization to improve the geometry of the peptide chain and remove all possible sterical clashes.

#### SMART-SEQ2 analysis

scRNA-Seq was performed in 384-well format. The tumors were isolated, rapidly processed, stained for a panel of surface markers and single cell sorted within approximately 90 minutes of organ harvest. In total 382 NK cells were sorted directly into 2 μL lysis buffer using a BD Influx from pooled tumors from either 3 WT and 3 *PD-1*^*−/−*^ KO mouse respectively. SMART-Seq2 libraries were prepared using the method described in Picelli et al. (2013) by the Eukaryotic Single Cell Genomics national facility at SciLife Laboratory, Stockholm.

Digital gene expression matrices were preprocessed and filtered using the Seurat v3.0 R package (https://github.com/satijalab/seurat). Outlier cells were first identified based on 3 metrics (library size, number of expressed genes, etc). Low abundance genes were removed by removing all genes that were expressed in less than 3 cells. The raw counts were normalized and transformed using the ‘LogNormalize’ function of Seurat. Highly variable genes were detected using the proposed workflow of the Seurat R package. Unsupervised clustering of the cells was performed and visualized in two-dimensional scatterplots via Uniform Manifold Projection (UMAP) function using the Seurat R package.

#### Microscopy and FCS analysis

##### Diffusion of PD-1 and PD-L1 on cell surface

Zeiss 510 microscope with a Confocor 3 system (Carl Zeiss Microimaging GmbH), C-Apochromat 40x/1.2 NA water objective was used for Fluorescence Correlation Spectroscopy (FCS) measurements (Vukojevic et al., 2008). Diffusion of interested molecules were measured using fluorescent labelled antibodies and FCS measurements were calibrated by measuring Alexa-488 and Alexa-647 dyes in solution at different power scale concentration whose diffusion coefficient is known. For cell preparation, spleens were isolated from from *RAG1*^−/−^ and *PD-1xRAG1*^−/−^ mice. From single cell suspension of splenocytes of mice, NK cells were isolated by MACS NK cell isolation kit mouse (Miltenyi Biotech Norden AB, Sweden). NK cells were stained for PD-1-Alexa flour 488 (RND systems) and PD-L1-Alexa flour 647 (RND systems), and microscopic chambers were coated with poly-L-lysine, so the cells are made to attach to the glass surface (Bagawath-Singh et al., 2016; Staaf et al., 2017). All the FCS measurements on cells were made on the cell surface for the diffusion of PD-1 and PD-L1.

##### FCS analysis

FCS Data was analyzed using MATLAB based written algorithm to have graphical user interface (GUI) for fitting. GUI permits to assume the initial fit coefficient like N-number of molecules, Tau D-Diffusion time for the molecule to diffuse within the focal volume, triplet state of the molecules. Different fit models and time fit domain was considered for free dyes and cells. Where 3D diffusion model fit was chosen for free dyes with time domain fit 0.5 μsecond to 0.1 millisecond and 2D diffusion model fit for cells with time fit between 1 millisecond to 5 second.

### Quantification and statistical analysis

All statistical analysis was performed using GraphPad Prism 9 software (La Jolla, CA). Data were presented as the mean ± SD. p value less than 0.05 is considered statistically significant. Significance is noted either in the text or figures.

## Data Availability

Smart-Seq2 data is available on Gene Expression Ominbus (GEO) with accession number GSE211488. Data reported in this article will be shared by the lead contact upon request. This article does not report original code. Any additional information required to reanalyze the data reported in this article is available from the lead contact upon reasonable request.
