# Supplementary material for: PD-1 expression on mouse intratumoral NK cells and its effects on NK cell phenotype
Source: iScience. 2022 Sep 16;25(10):105137. doi: 10.1016/j.isci.2022.105137 (PMC9523278; doi:10.1016/j.isci.2022.105137)
Supplement: Document S1. Figures S1–S10 and Tables S1 and S2 [file mmc1.pdf]

iScience, Volume *25*

Supplemental information

PD-1 expression on mouse intratumoral NK cells and its effects on NK cell phenotype

Arnika K. Wagner, Nadir Kadri, Chris Tibbitt, Koen van de Ven, Sunitha Bagawath-Singh, Denys Oliinyk, Eric LeGresley, Nicole Campbell, Stephanie Trittel, Peggy Riese, Ulf Ribacke, Tatyana Sandalova, Adnane Achour, Klas Kärre, and Benedict J. Chambers

Supplemental Information

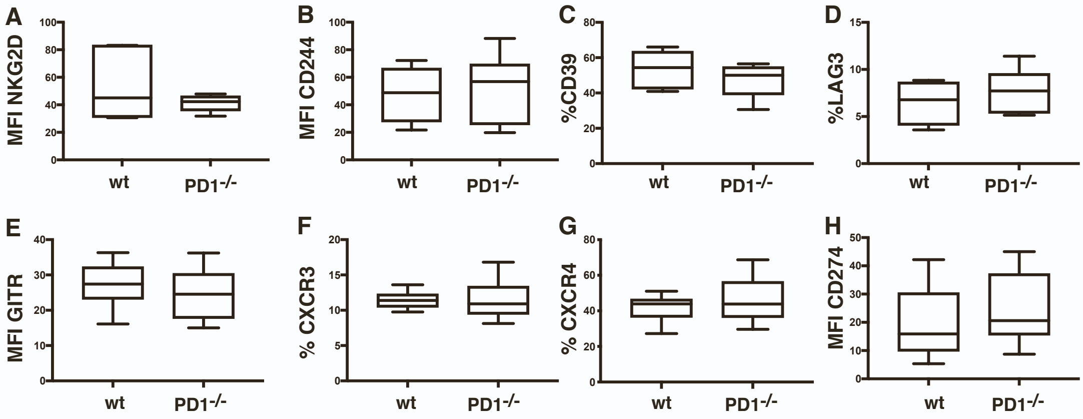

Supplemental Figure S1. Expression of NK cell markers and chemokine receptors on wt and PD-1$^{-/-}$ NK cells, related to Figure 1. A, Percent of NK cells expressing NKG2D, B, MFI of CD244, C, percent of CD39, D, percent of LAG3, E, MFI of CD274 F, MFI of GITR G, percent of CXCR3 and H, of CXCR4.

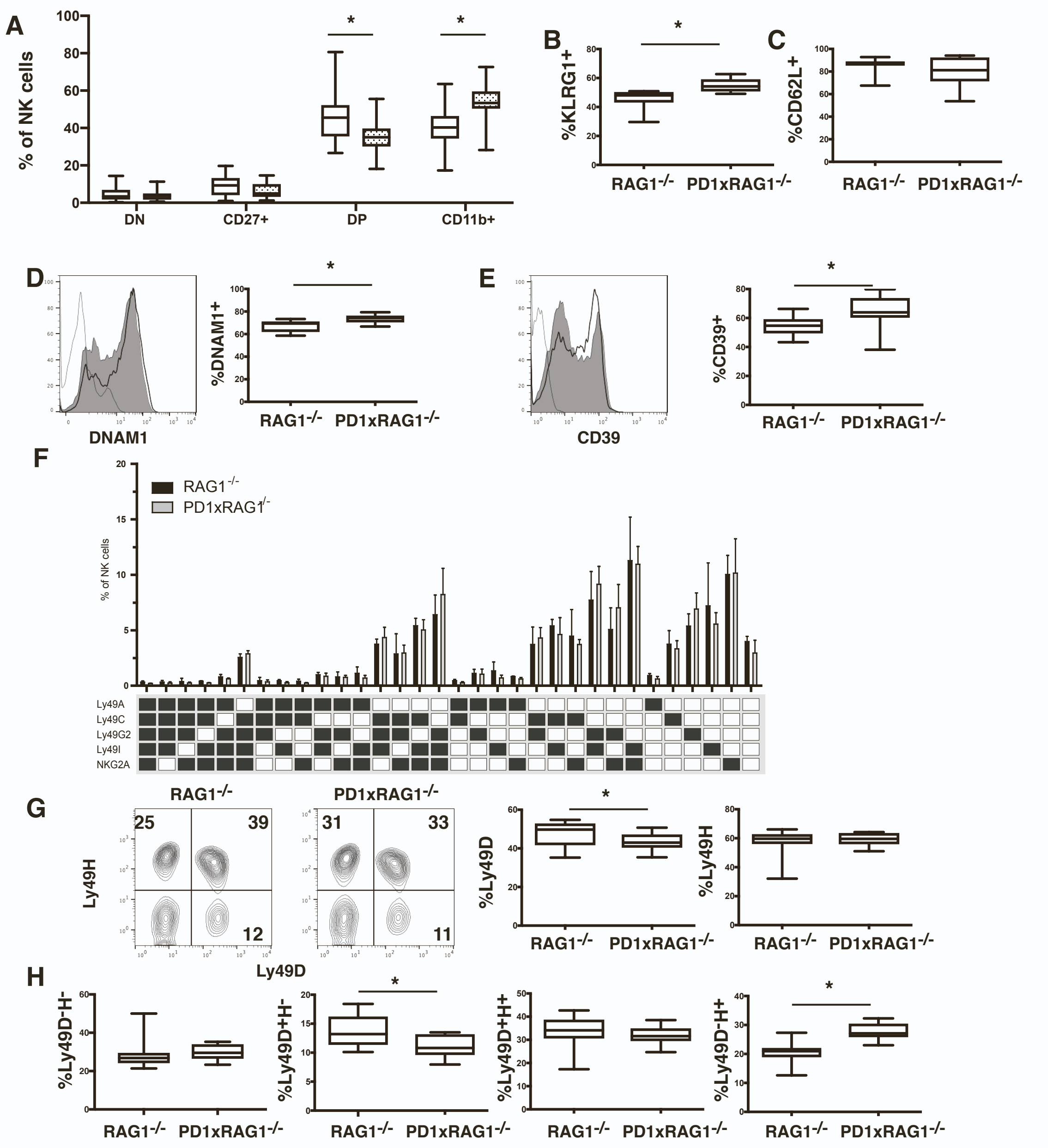

Supplemental Figure S2. Phenotype of NK cells from *RAG1$^{-/-}$* and *RAG1$^{-/-}$PD-1$^{-/-}$* mice, related to Figure 1. A, Expression of CD11b and CD27 on NK cells from *RAG1$^{-/-}$* (open bars) and *RAG1$^{-/-}$*PD-1$^{-/-}$ mice (shaded bars) (*p<0.01 MannWhitney test, n=14 mice, shown as mean±SD). B, Expression of KLRG1 on NK cells from *RAG1$^{-/-}$* and *RAG1$^{-/-}$PD-1$^{-/-}$* mice (*p<0.01 MannWhitney test, n=14 mice shown as mean±SD). C, Expression of CD62L on NK cells from *RAG1$^{-/-}$* and *RAG1$^{-/-}$PD-1$^{-/-}$* mice. D, Expression of DNAM-1 on NK cells from *RAG1$^{-/-}$* and *RAG1$^{-/-}$PD-1$^{-/-}$* mice, bar graphs represent percent expressing cells and the mean floursecent intensity of expression (*p<0.01 MannWhitney test, n=14 mice shown as

mean±SD). E, Expression of CD39 on NK cells from *RAG1*$^{-/-}$ and *RAG1*$^{-/-}$*PD-1*$^{-/-}$ mice, bar graphs represent percent expressing cells and the mean floursecent intensity of expression (*p<0.01 MannWhitney test, n=14 mice shown as mean±SD). F, Expression of inhibitory Ly49 molecules and NKG2A on NK cells from *RAG1*$^{-/-}$ (open bars) and *RAG1*$^{-/-}$*PD-1*$^{-/-}$ mice (closed bars) (*p<0.01 Mann Whitney). G, Expression of activating Ly49 molecules on NK cells from *RAG1*$^{-/-}$ and *RAG1*$^{-/-}$*PD-1*$^{-/-}$ mice. (*p<0.01 MannWhitney test, n=14 mice H, Expression of Ly49D and Ly49H populations on NK cells from *RAG1*$^{-/-}$ and *RAG1*$^{-/-}$*PD-1*$^{-/-}$ mice (*p<0.01 MannWhitney test, n=14 mice, shown as mean±SD).

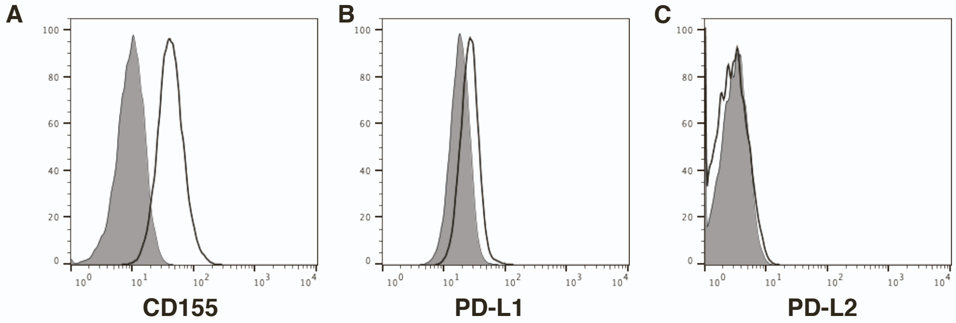

Supplemental Figure S3. Expression of CD155, PD-L1 and PD-L2 on MTAP1A tumor cells, related to Figure 2. MTAP1A tumor cells were stained with mAbs specific for A, CD155, B, PD-L1 , or C, PD-L2 and analyzed by flow cytometry for expression of these markers. Grey histograms represent unstained cells while white histograms represent cells stained with the respective antibody.

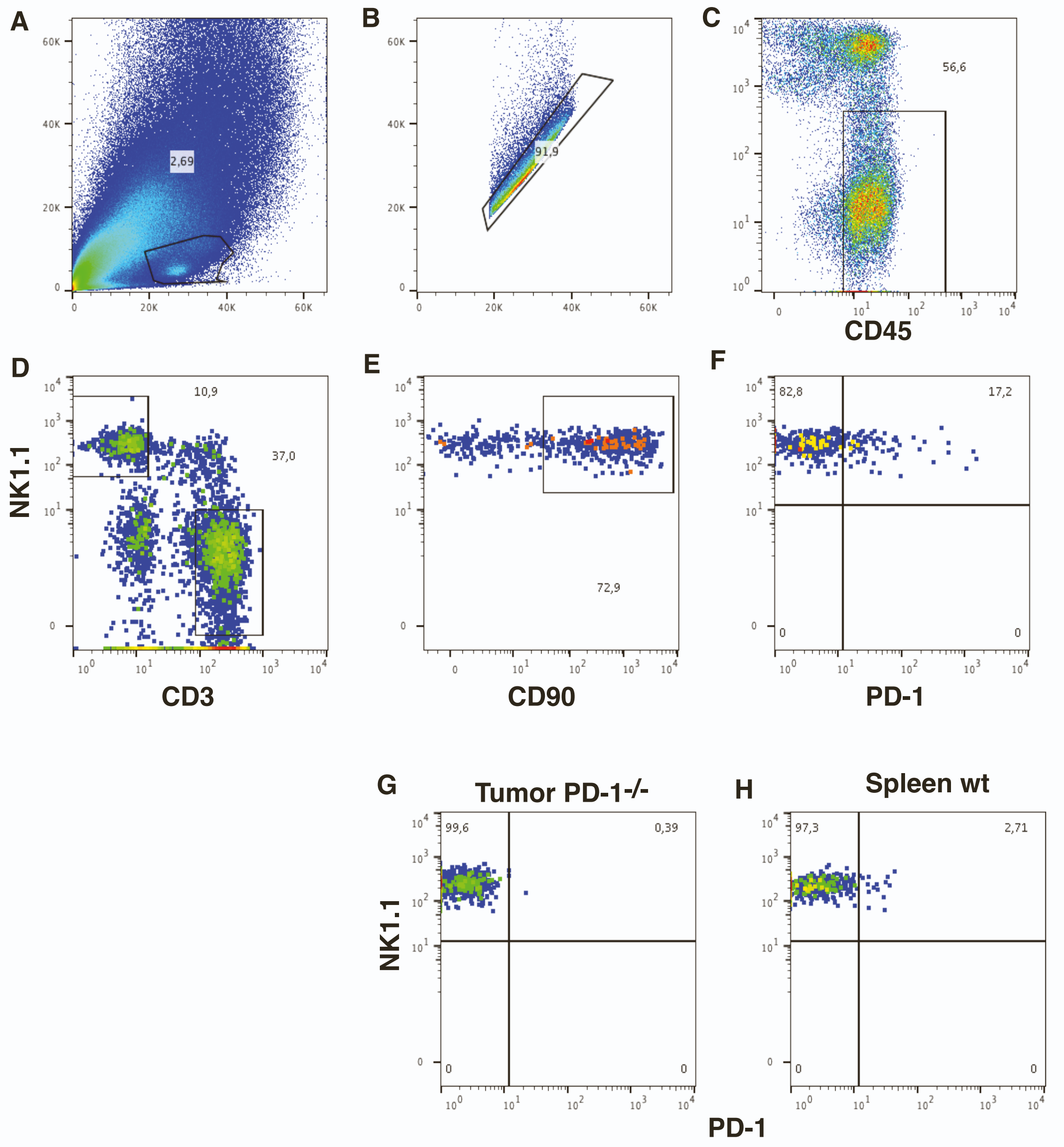

Supplemental Figure S4. Gating strategy for intratumoral NK cells, related to Figure 2. MTAP1A tumors were excised, crushed and filtered to get a single cell suspension. Flow cytometric analyses (A-B) of markers for leukocytes C, NK cells and CD3 D-F were performed. G, PD-1 expression was assessed on intratumoral NK cells in *PD-1*$^{-/-}$ mice and H, on spleen NK cells of wt mice.

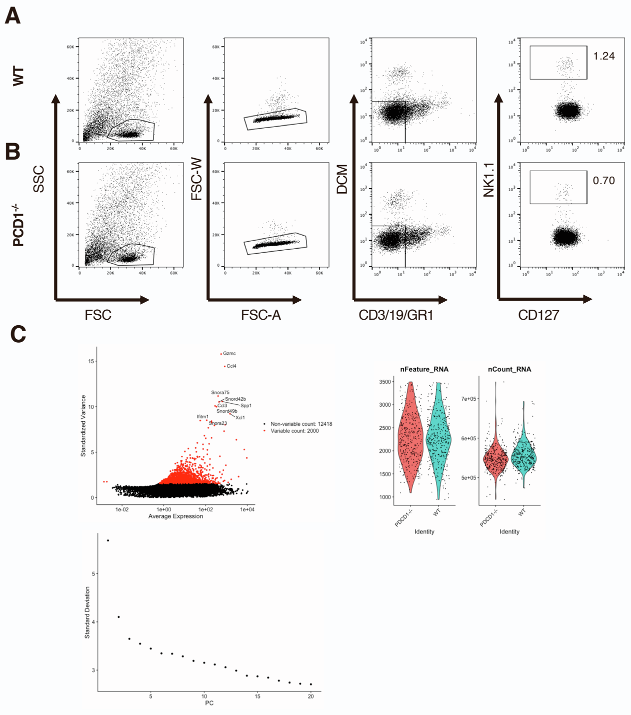

Supplemental Figure 5. Sorting strategy and scRNA-SEQ QC, related to Figure 3. Viable NK cells (NK1.1+CD3- CD19-GR1-) were sorted from pooled tumors from either WT (a) or *PDCD1*$^{-/-}$ (PD-1$^{-/-}$) (b) according to the gating strategy shown. (c) SMART-SEQ2 data was processed according to the standard Seurat v3 pipeline with an elbow plot used to determine cutoff for dimensionality reduction. The data was processed according to the standard Seurat v3 pipeline. A total of 18 cells failed to pass the QC and were removed from analysis.

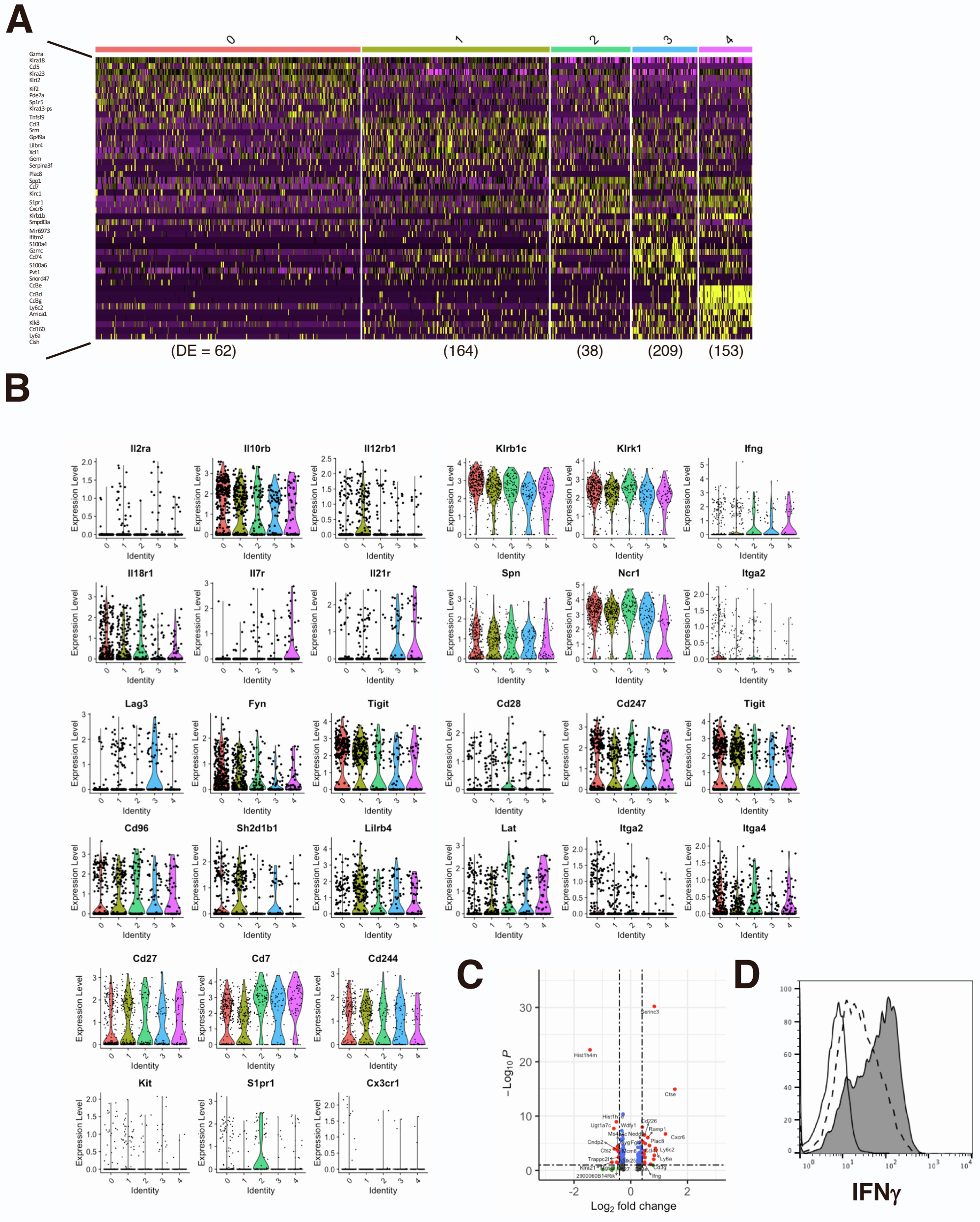

Supplemental Figure 6. Extended analysis of scRNA-Seq of inflitrating WT and *PD-1*-/- NK, related to Figure 3. (A) Heatmap visualisation of top 10 genes per cluster. Number of DE genes

per cluster is shown below. (B) Violin plots showing expression of the genes Il2ra, Il10rb, Il12rb1, Tnf, Ifng, Csf1, Il18r1, Il7r, Il21r, CD44, Ly6c2, Ly6a, Lag3, Fyn, Tigit, CD28, CD247, CD96, Sh2d1b1, Lilrb4, Lat, Itga2, Itga4, Klrb1, Klf3, Ccr7, Itgb2, Cd9, CD160, CD5, CD244, Nfil3, Anxa1, S1pr5 and B4galnt1, where expression significantly differs between WT and *PD-1*$^{-/-}$ NK cells. (C) Volcano plot depicting genes upregulated in WT (to the left) and *PDCD1*$^{-/-}$ (to the right) NK cells. Intracellular levels of IFNγ following cytokine stimulation of NK cells from (dashed line) WT mice and (shaded line) *PD-1*$^{-/-}$ mice (representative plot from three experiments). (D) IFNγ levels in NK cells from wt (dashed line) and *PD-1*$^{-/-}$ mice (shaded line), following stimulation with IL-12,IL-15 and IL-18.

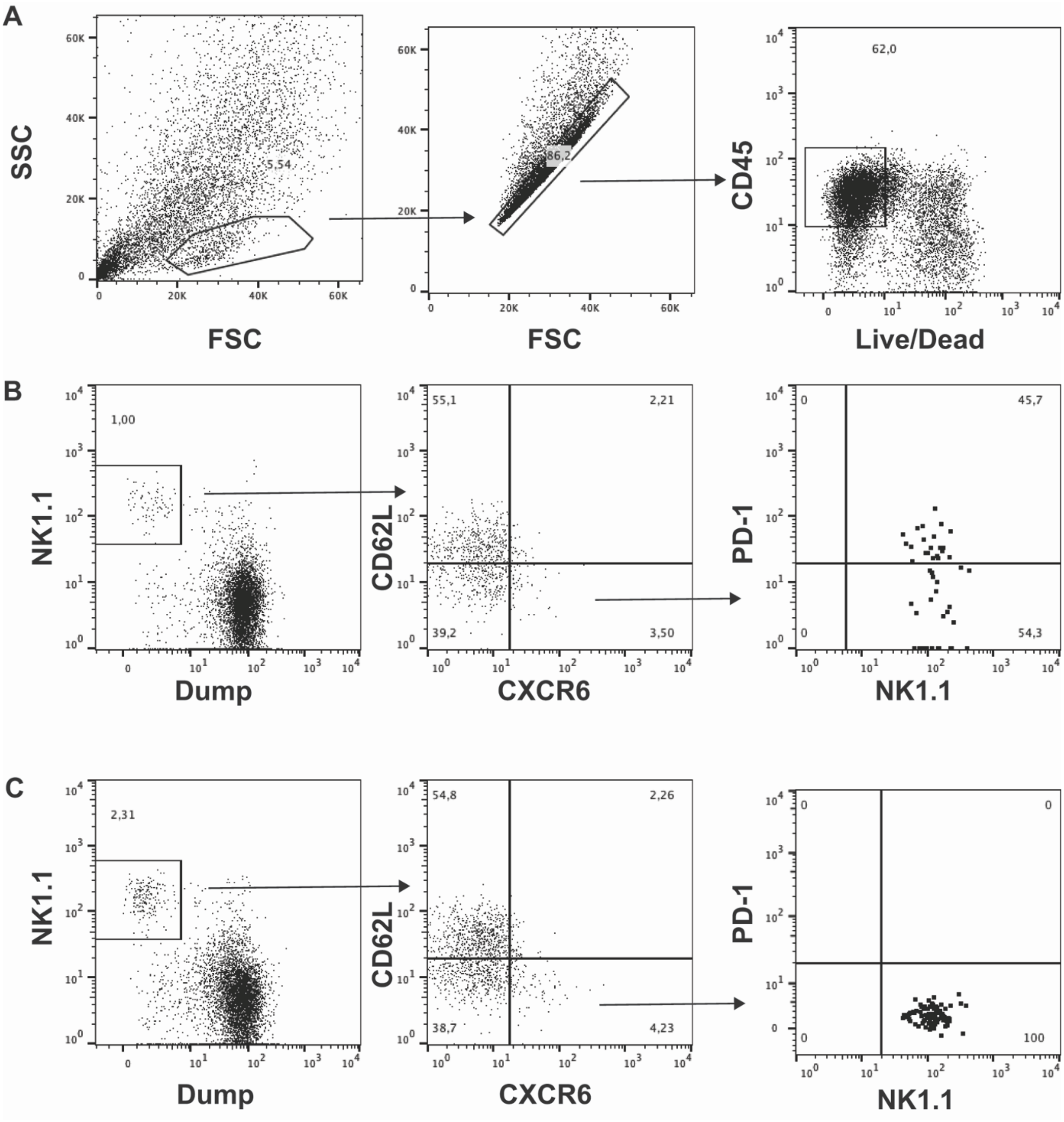

Supplemental Figure 7. Gating strategy for intratumoral CXCR6^{+} NK cells, related to Figure 4. MTAP1A tumors were excised, crushed and filtered to get a single cell suspension. Flow cytometric gating (A) of lymphocytes from tumors (B), gating on wildtype NK cells and their expression CXCR6 and CD62L and finally expression of PD-1 on CXCR6^{+} NK cells (C) gating on PD-1$^{-/-}$ NK cells and their expression CXCR6 and CD62L and finally expression of PD-1 on CXCR6^{+} NK cells.

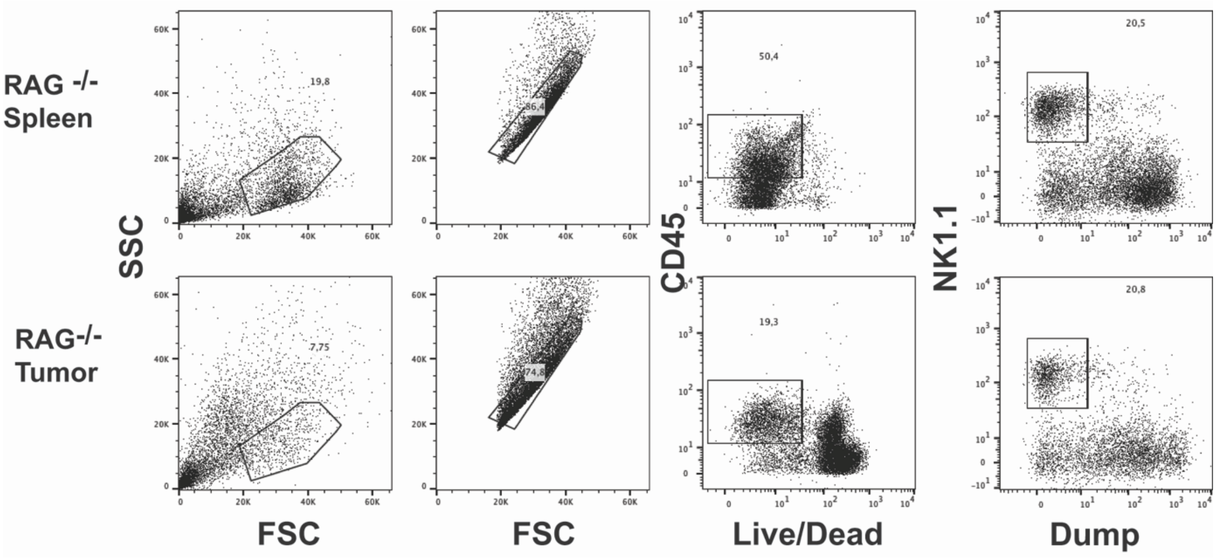

Supplemental Figure 8. Gating strategy for intratumoral CXCR6^{+} NK cells on RAG-1$^{-/-}$ mice, related to Figure 4. Splenocytes and MTAP1A tumors were excised, crushed and filtered to get a single cell suspension. Flow cytometric gating of splenic lymphocytes and from tumors.

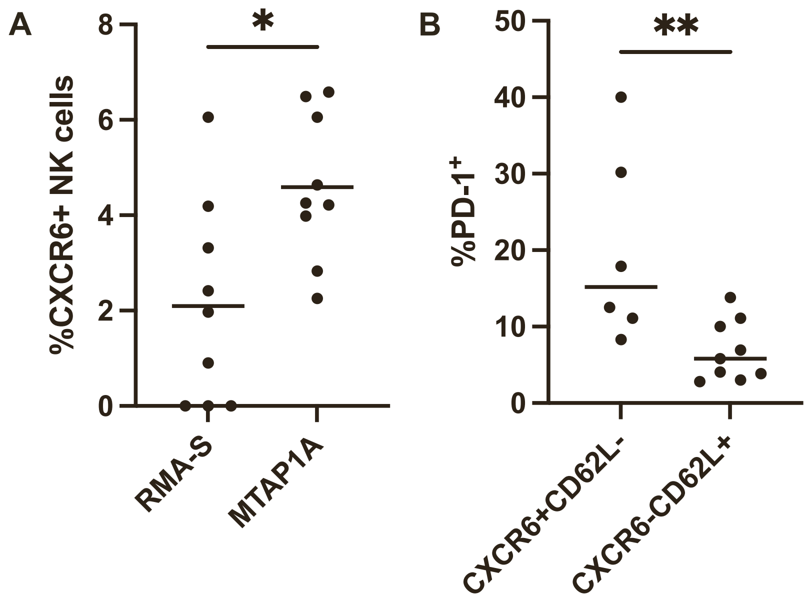

Supplemental Figure 9. Frequency of $CXCR6^+$ NK cells from MTAP1A and RMA-S tumors, related to Figure 4. A. Percentage of $CD62L^-CXCR6^+$ NK cells recovered from RMA-S and MTAP1A (*p<0.05 Mann-Whitney test n=9 mice). B. Frequency of PD-1 on $CXCR6^+$ tumor infiltrating NK cells in mice inoculated with RMA-S (**p<0.05 Mann-Whitney test n=6-9 mice).

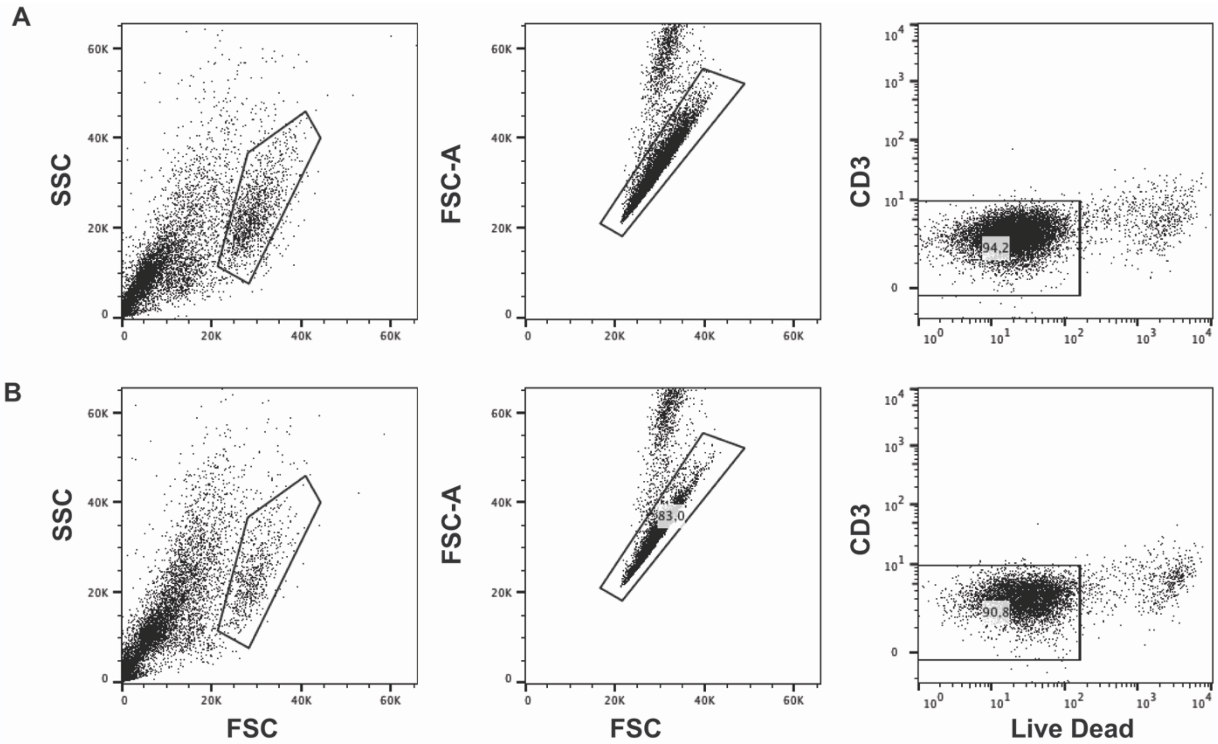

Supplemental Figure 10. Gating strategy for in vitro derived NK cells, related to Figure 5. NK cells were isolated using negative sorting beads for NK cells (Miltenyi Biotech) and cultured in vitro with IL-12 IL-15 and IL-18. (A) B6 NK cells (B) PD-1$^{-/-}$ NK cells. No T cell contamination could be observed in these cultures.

Supplemental Table 1: P values of genes that significantly upregulated in PDCD1-/- NK cells, related to Figure 3

Gene	*p-value*
4833420G17Rik	0.0294560089322125
Aarsd1	0.00014546275752589
Ablim1	0.00244304042156039
Ap4s1	0.0354143529581902
Bscl2	0.0104518050444323
Capg	0.000754405873530379
Ccl4	0.00169530836212815
Cd160	0.00376332357863304
Cd226	3.13682908855125e-07
Cd3e	0.0450593340578062
Comt	0.0045328912944519
Coro2a	5.6699407468564e-05
Ctse	1.15768218666942e-15
Cul1	0.0299923586585598
Cxcr6	1.97343268509504e-07
Cysltr2	0.0051475976240368
D230025D16Rik	0.0121353965816209
Dusp2	0.000255515137305345
Eif2d	0.00525748204514534
Elovl1	0.0268176741044549
Evi2a	0.0163727636886736
F2r	0.010761567739447
Fbxl6	0.00121923709570013
Fgl2	1.13557269866248e-05
Gimap7	0.00837115833431172
Glrx	0.00351160751773035
Gns	0.00276166589598885
Got1	0.0153115467295851
Gzmb	0.00444052027700713
Hibadh	0.00478246029204708
Hnrnpm	0.03914043719802
Id2	8.32189052874474e-06
Igsf5	0.0110773911622674
Itga1	5.66891895628475e-05
Klhl25	0.0280228106909095
Klrb1b	0.000215424070178674
Klrc1	0.000906898344562637
Klrg1	0.00898488940611906
Lag3	0.00414929516522654
Lgals3	0.000955605594016018

Ly6a	0.000181160676017526
Ly6c2	9.21126370197399e-05
Mapk14	4.34626898068145e-05
Mgat2	0.0177035749633055
Micu2	0.0173752833982563
Mlycd	0.0163355758753552
Mmadhc	0.0378199706460093
Msto1	0.0162227278120772
Nadk	0.0110599838100958
Naga	0.00206253123672797
Ndufs1	0.00644427838415626
Nedd9	5.00263546868797e-06
Nmi	0.0107098645852247
Nup93	0.0483394265336457
P4hb	0.0430787925200768
Plac8	2.6879413217007e-05
Ptplad1	0.0361559828741849
Ramp1	9.29433013791078e-07
Rassf5	0.00116735607946277
Rbks	0.0109825538550385
Rinl	0.0135157309931973
S100a4	0.0138118915050287
Sdcbp	0.00240501003710846
Serinc3	6.27677503191847e-31
Sh3bp1	0.00804558051687257
Sla	0.0169531313452193
Slc2a3	0.00447502894047429
Snora28	0.0326539042092242
Spp1	0.00523172242147817
Srgn	0.00052963243923553
St6galnac4	0.00231663645736426
Stk17b	0.00936945039904389
Styk1	0.00256774322556803
Tmem128	0.00109479524498518
Tmem176b	0.0303113229640398
Tmem199	0.00410912503961128
Tmem37	0.0126432798822501
Tspan3	0.0327594540096517
Txk	0.00762086135431145
Ubap2l	0.0440531980453362
Uckl1	0.0145827552001765
Ugp2	0.0189624723669044
Utp11l	0.0394799506167981

Vmp1	0.00041638049673073
Wdfy1	1.06702627915021e-08
Zc3h14	0.0138918531386612
Zfp36	0.00221040436852507
Zfp692	0.0447096092458944

Supplemental Table 2: P values of genes that significantly upregulated in WT NK cells, related to Figure 3

0610037L13Rik	*0.0479300706665614*
1700020I14Rik	0.0453053177288735
2900060B14Rik	0.0404041545808012
4930486L24Rik	0.000400511875558075
9030624J02Rik	0.000372202938019854
Anapc2	0.0163699729204511
As3mt	0.0011811244062903
Cd300lf	0.000806216994065812
Cd9	0.00265512708781193
Cdc37	0.00482981586245807
Cmc1	0.0201607924039511
Cndp2	0.000168918497256701
Cnot8	0.00504607558641687
Ctsz	0.000110090159134527
Cxxc1	0.00830250056366626
Cyba	0.00011125386178722
Dctn3	0.0305564867562836
Eif3i	0.0195006719410028
Fam195b	0.0494070760359527
Fez2	0.00636673256680017
Fis1	0.00636636625757695
Glrx5	0.0228145183424616
Gyg	0.000336207620171877
Hemgn	0.000517787304179204
Hist1h1e	1.11246146661204e-09
Hist1h4c	0.0281021955167639
Hist1h4m	6.55865752557258e-23
Ict1	0.0259951334917959
Ifi27	0.00474219709535523
Irf5	0.000323659704029428
Jun	0.000285056927056873
Kcnip3	0.00210098602235471
Klra13-ps	0.0331683991053854
Klra21	0.0307030769116372
Lgals1	1.80274751617846e-06

Lta4h	0.00136166904492055
Mcm6	0.000466756919579111
Mgst2	0.0307433799133878
Mrps2	0.0265829764333398
Ms4a4c	3.89740981356844e-05
Nck1	0.000785343675388053
Nfkb2	0.00384483049748892
Nit2	0.0276830640719862
Nme2	0.0184543834576273
Nsmce1	0.00678835592878754
Padi2	0.000736394481885643
Park7	0.0251050167644584
Pglyrp1	0.00642580437177118
Psmb6	0.00666401481564645
Rnpep	3.54770508638786e-06
Rpl13a	4.33451940567619e-11
Rpl32	4.9862296394884e-08
Rpl41	5.91528715064592e-05
Rps26	1.84934669797884e-06
Rps5	3.75693292047592e-07
Rps9	1.50640362629844e-06
Rrad	0.0146622016496049
Serpina3g	0.0023819709241389
Slc25a4	0.0126756121714561
Slc2a6	0.0109447616261956
Smn1	0.00995191442154229
Stk25	0.00219701812850478
Tcf7	9.15486885025558e-05
Tmem80	0.0342975349694778
Tmsb10	4.88414805761391e-11
Toe1	0.0288994213097058
Trappc2l	0.00383832466555109
Tsnax	0.0136314433969804
Tssc4	0.0298338221057074
Tyrobp	2.29393142858282e-05
Ugt1a7c	1.95929659888963e-08
Vps41	0.0382164230587772
Xcl1	0.0215149029319816
